# Evaluation of an Online-Based Self-Help Program for Patients With Panic Disorder: Randomized Controlled Trial

**DOI:** 10.2196/54062

**Published:** 2025-04-02

**Authors:** Christopher Lalk, Teresa Väth, Sofie Hanraths, Luise Pruessner, Christina Timm, Steffen Hartmann, Sven Barnow, Julian Rubel

**Affiliations:** 1 Clinical Psychology and Psychotherapy of Adulthood Institute of Psychology University Osnabrück Osnabrück Germany; 2 Clinical Psychology and Psychotherapy Institute of Psychology Heidelberg University Heidelberg Germany

**Keywords:** internet-based CBT, agoraphobia, well-being, iCBT, internet-based intervention, panic disorder with and without agoraphobia, panic disorder, self-help, quality of life, effectiveness, online, self-help intervention, panic symptoms, well-being, daily functioning

## Abstract

**Background:**

Panic disorder is an anxiety disorder marked by severe fear of panic attacks in the absence of causes. Agoraphobia is a related anxiety disorder, which involves fear and avoidance of specific situations in which escape or help may be difficult. Both can be debilitating and impair well-being. One treatment option may be internet-based cognitive behavioral therapy (iCBT), which allows large-scale application and may overcome treatment barriers for some individuals.

**Objective:**

This study aimed to evaluated the effectiveness of a novel online self-help intervention for panic disorder with or without agoraphobia. As our primary hypotheses, we expected the intervention to improve panic and agoraphobia symptoms and well-being. Our secondary hypotheses entailed improvements in daily functioning, mental health literacy, working ability, and health care use in the intervention group.

**Methods:**

German-speaking patients (N=156) aged 18-65 years with internet access and a diagnosis of panic disorder with or without agoraphobia were recruited for this randomized controlled trial. The intervention group (n=82) received access to a 12-week online self-help program entailing psychoeducation, cognitive restructuring, exposure, and mindfulness elements. The control group (n=72) received care as usual during the study period and was offered the prospect of using the program after 12 weeks. The primary outcomes were assessed via the Panic and Agoraphobia Scale (PAS) and the WHO (World Health Organization)-5 Well-Being Index (WHO-5). Mixed effect models were computed using multivariate imputation by chained equation for the analysis of intervention effects.

**Results:**

In the intervention group, participants completed on average 7.3 out of 12 (60.8%) modules, and 27 out of 82 (32.1%) participants finished the whole course. Changes in PAS revealed a significant effect in favor of the intervention group (*t*_110.1_=–2.22, *P*_adj_=.03) with a small to moderate effect size (*d*=–0.37, 95% CI –0.70 to –0.04). No significant effect was found for the second primary outcome WHO-5 (*t*_149.8_=1.35, *P*_adj_=.09) or the secondary outcomes. Improvements were observed in anxiety (*t*_206.8_=–4.12; *P*<.001; Cohen *d*=–0.60, 95% CI –0.089 to –0.32) and depression (*t*_257.4_=–3.20; *P*<.001; Cohen *d*=–0.41 95% CI –0.66 to –0.16). No negative effects were associated with the intervention (*t*_125_=–1.14, *P*=.26).

**Conclusions:**

The presented online intervention can help reduce the core symptomatology of panic disorder and agoraphobia, as well as anxiety symptoms and associated depression. No effects were found for well-being and secondary outcomes. This may be due to higher illness burden in the intervention group and possibly the COVID pandemic, which caused unique challenges to patients suffering from panic disorder. Therefore, further research and intervention adaptations may be warranted to improve these outcomes.

**Trial Registration:**

German Clinical Trials Register DRKS00023800; https://drks.de/search/en/trial/DRKS00023800

## Introduction

### Background

Panic disorder involves recurring sudden panic attacks and a constant fear of experiencing more episodes. These attacks come with physical symptoms like breathing issues, palpitations, sweating, and nausea, as well as psychological symptoms such as derealization and a fear of losing control or dying [1,2]. Agoraphobia, which affects 35%-65% of people with panic disorder [3], involves excessive fear and avoidance of situations where escape may be difficult, like public transport, and help may not be readily available in case of a panic attack. Both panic disorder and agoraphobia are closely related and often seen as part of a continuum [4]. In earlier versions of the *DSM* (*Diagnostic and Statistical Manual of Mental Disorders*) (ie, the third and fourth editions [DSM-III and DSM IV]) agoraphobia was defined as a feature of panic disorder (ie, panic disorder with agoraphobia or panic disorder without agoraphobia), which is why some research is organized around this conceptualization.

Panic disorder, with or without agoraphobia, comes with functional impairments and reduced well-being, increasing the risk of other mental disorders [5-8]. It also imposes a significant economic burden, surpassing that of other anxiety, mood, or alcohol-related disorders [9]. These costs include hospital treatments, health care visits, and, most notably, absenteeism, which accounts for 60% of all expenses [10]. Thus, it is crucial to offer effective and timely treatment for panic disorder for both societal and individual well-being.

Regarding psychotherapeutic treatments, recently cognitive behavioral therapy (CBT) and short-term psychodynamic therapy have been identified as treatments of choice [11]. CBT targets fear and avoidance behavior by psychoeducation, exposure therapy, cognitive restructuring, mindfulness, and acceptance interventions leading to large effect sizes compared with a waitlist group (hedges *g*=0.96; [12]). Also, effects remain superior to treatment as usual for 6 months of follow-up [13]. Psychopharmacology yields small to moderate effect sizes in comparison to placebo and similar to CBT [14]. Although, compared with pharmacotherapy, CBT shows longer-term treatment effects, better cost-efficacy, and higher patient acceptance [15].

An analysis of treatment barriers in anxiety disorders [16] showed that 63 out of 77 (81.8%) patients with panic disorder contemplate treatment. Still, only 40 out of 59 (67.3%) sought help at least once in their life, and this number was even lower in patients with agoraphobia (21/56, 36.9%). Frequently reported barriers to help-seeking include self-reliance, presumed ineffectiveness, high waiting periods, or problems with the practitioner [16]. Further, negative attitudes and lack of knowledge and appropriate beliefs about mental health are associated with less help-seeking behavior [17].

During treatment waiting periods, it is recommended to offer patients self-help programs based on CBT [18], since technology-based treatment alternatives can help surpass the aforementioned barriers [19]. Most of these alternatives are based on CBT, since it is well suited for online intervention delivery due to its highly structured, directive, standardized nature and its focus on psychoeducation and homework [20]. Of particular note is the benefit of internet-based CBT (iCBT) to the healthcare system, as it is a cost-effective treatment alternative with similar efficacy to face-to-face CBT [21,22]. Also, iCBT can help to bridge the waiting periods for face-to-face psychotherapy, which on average lasts several months in Germany and has further increased since the beginning of the COVID-19 pandemic [23,24].

A systematic review and meta-analysis including 27 studies on iCBT for panic disorder [25] showed high efficacy and effectiveness for reducing symptoms of panic disorder (hedges *g*=1.16) and agoraphobia (hedges *g*=0.91) compared with waitlist. Another meta-analysis of 13 RCTs for panic disorder and agoraphobia found no efficacy difference between unguided iCBT and face-to-face CBT in terms of panic and agoraphobia symptoms, comorbid depression and anxiety, as well as quality of life improvement [26] indicating that unguided iCBT may be comparably effective to face-to-face CBT. However, in a network meta-analysis of 74 efficacy trials, unguided iCBT was not superior to care as usual for the treatment of panic with or without agoraphobia [27]. In the same analysis, both guided forms of iCBT and face-to-face CBT were superior to care as usual, though not superior to unguided iCBT.

Therefore, results regarding the efficacy and effectiveness of unguided iCBT for panic and agoraphobia are mixed, paradoxically indicating similar effects as face-to-face CBT while also indicating no improvement to care as usual. Also, little research can be found investigating broader effects on well-being, functioning, mental health literacy, working ability, and health care use. These seem particularly relevant to assess effects on cost-effectiveness.

### Objectives

This study aimed to evaluate a 12-week online self-help program (Selfapy) for patients with panic disorder with or without agoraphobia within the German health care system. For the analysis, we had 2 objectives: first, we compared the intervention with care as usual, since the superiority for unguided self-help toward care as usual has been questioned; second, we assessed effectiveness for a range of broader outcomes with limited research data (eg, well-being and functioning).

### Hypotheses

Based on previous research, the primary hypotheses expect superior improvement on panic and agoraphobia symptoms and well-being in the intervention group (IG). The secondary hypotheses expect superior improvement of daily functioning, work capacity, mental health literacy, and the efforts and burdens of patients for the health care sector. Finally, changes in comorbid anxiety and depression symptoms as well as negative effects of the program beyond symptomatology, are being explored as exploratory hypotheses. All outcomes were tested against the control group (CG) after 12 weeks.

## Methods

### Study Design

The study is reported according to the CONSORT-EHEALTH (Consolidated Standards of Reporting Trials of Electronic and Mobile Health Applications and Online Telehealth) eHealth guidelines (Multimedia Appendix 1). The parallel group trial was preregistered beforehand [28] and a study protocol was published [29]. Eligible patients were randomly assigned to the IG or CG in a 1:1 ratio. Patients in the IG could access the intervention immediately after randomization, while the CG could only access the intervention after a waiting period of 12 weeks. Interim and final evaluations occurred 6 (T2) and 12 (T3) weeks after the baseline assessment (T1).

### Participants

Announcements for study participation were published in the whole of Germany via a university email newsletter, social media, and on flyers in clinics, pharmacies, and practices of medical doctors and psychotherapists. After an online prescreening, participants were invited to choose an appointment for a remote diagnostic interview assessing the inclusion and exclusion criteria. To this end, a structured diagnostic interview diagnostisches interview psychischer störungen-open access (DIPS-OA, [30,31]) was conducted with every participant via video calls. Regarding *DSM-IV-TR* (*Diagnostic and Statistical Manual of Mental Disorders* [Fourth Edition, Text Revision]) criteria, the DIPS-OA was found to have acceptable interrater (0.78) and retest (0.76) reliability for anxiety disorders [32]. Altogether 4361 people started the online screening, of whom 764 (17.5%) were deemed eligible to be scheduled for the diagnostic interview. The online screening consisted of short screening questionnaires of the inclusion and exclusion criteria.

### Inclusion and Exclusion Criteria

Video calls were conducted with all individuals who checked the prescreening criteria to assess inclusion and exclusion criteria, during which eligibility was assessed via the DIPS-OA. Trained psychologists conducted all interviews under the supervision by a certified psychotherapist (CBT).

Eligible individuals were those who (1) were between 18 and 65 years of age, (2) had sufficient knowledge of the German language, (3) had uninterrupted internet access, (4) provided electronic informed consent to participate in the study, and (5) met the criteria for a diagnosis of panic disorder with or without an additional diagnosis of agoraphobia.

Individuals were excluded if they did not meet any of the inclusion criteria or met any of the following criteria: (1) past or current diagnosis of bipolar disorder, (2) past or current diagnosis of psychotic disorder, (3) current diagnosis of substance dependence, (4) current diagnosis of a severe major depressive episode, and (5) acute suicidality. The criteria were chosen because they could interfere with the successful implementation of the course.

### Intervention

The online self-help program for the treatment of panic disorder with or without agoraphobia (Selfapy) was conducted as the intervention. The program is based on evidence-based methods and exercises derived from CBT (such as psychoeducation, cognitive restructuring, or exposure), as well as elements from Mindfulness-Based Therapy (eg, [33,34]). The online intervention consists of core modules, which include mandatory exercise content, and a subsequent set of optional specialization areas that are individually selectable (for a complete overview, [29]). Each module covers a specific topic, such as exposure, mindfulness, or problem-solving training. The modules contain informative texts, videos, audio, and interactive exercises and can be used via the web as well as on mobile devices.

Participants completed the online program independently and without support. They were monitored for suicidality in which case they were messaged by a psychologist. Active communication only occurred for safety reasons. The IG had immediate access to the 12-week self-help treatment and were advised to spend 15-20 minutes daily on it.

### The CG

The CG received no treatment for 12 weeks but could seek other assistance beyond this trial, such as medication or therapy, to mimic routine care. All concurrent treatments were self-reported. The CG received the study intervention after study completion (=after 12 weeks), since 12 weeks is a common waiting time for psychotherapy in Germany [24].

### Outcomes

Measures were conducted at 3 different points in time: Before the start of the intervention (T1, baseline), after 6 weeks (T2, during treatment), and 12 weeks after the beginning of the intervention (T3, post treatment). At each measurement time point, the primary, secondary and exploratory outcomes were assessed. Assessment was conducted via an online assessment platform [35].

#### Primary Outcome Measures

The change in panic and agoraphobia symptoms was evaluated using the Panic and Agoraphobia Scale (PAS; [36]). The PAS consists of 13 items that are rated on a 5-point Likert scale. There scale contains 5 subscales: panic attacks, agoraphobic avoidance, anticipatory avoidance, disability, and worries about health. In our data, we calculated McDonald ω [37] as a reliability measure with ω=0.86 at T1. Well-being was assessed by the WHO (World Health Organization)-5 Well-Being Index (WHO-5; [38]). The WHO-5 contains items measuring positive mood, calmness, high energy levels, good rest, and interest in daily activities.

#### Secondary Outcome Measures

Functioning in daily life was measured by the Work and Social Adjustment Scale (WSAS; [39]) assessing professional and personal functioning. Reliability was good (ω=0.79) at T1.

Work capacity was measured with the iMTA Productivity Cost Questionnaire (iPCQ) to assess the amount of lost working hours in the last 4 weeks due to absenteeism or distress-related impaired work capabilities (iPCQ [40]).

Mental health literacy was measured with the Mental Health Literacy Scale (MHLS; [41]) with high reliability (ω=0.85 at T1).

The extent of therapy-related efforts and burdens of patients (Client Sociodemographic and Service Receipt Inventory [CSSRI] [42]) was collected on three subscales: CSSRI-partly inpatient to assess partly inpatient treatment, CSSRI-complementary to assess complementary services (eg, self-help groups), and CSSRI-ambulant to assess outpatient services (eg, psychotherapy treatment and medical treatment).

#### Additional Outcome Measures

Adverse treatment effects were assessed with the Negative Effects Questionnaire (NEQ; [43]). The NEQ contains 32 items and showed high reliability (ω=0.89 at T3). Also, general symptoms of anxiety were assessed using the Beck Anxiety Inventory (BAI; [44]) with ω=0.89 at T1. Depressive symptoms were collected with the Patient Health Questionnaire-9 (PHQ-9 [45]; ω=0.74) at T1.

### Sample Size

The between-group effect size estimate was based on meta-analytic evidence for effect sizes in unguided online psychological interventions for anxiety disorders Cohen (*d*=0.45; eg, [46]). This effect was used as the basis for sample size determination. For the planned mixed model with 2 measurement time points with a general correlation structure [47], a directed hypothesis, a group allocation of 1:1, a power of 0.80, and an α level of .025 after Bonferroni-Holm correction, a total of 156 patients (78 per group) were needed. The number of cases was calculated using the *R*-tool (Michael C. Donohue) longpower [48]. For the secondary outcomes, we calculated a minimal detectable effect size of Cohen *d*=–0.46 with 80% power and an α level of .0125 (Bonferroni-Holm adjustment) based on a post hoc power analysis of the WSAS with simr (Peter Green) [49]*.*

### Randomization and Blinding

Randomization in 1:1 ratio without stratification or blocks was done automatically by a computer-generated code. Participants were automatically informed of their group via email. Data collection, evaluation, and statistical analysis were carried out blindly. A team member not involved in the analysis coded the group variable, and analysis scripts were prepared before knowing the actual data.

### Statistical Analyses

The statistical analyses were conducted following the study protocol [29]. The analyses were performed with R (version 4.2.0; R Core Team) [50].

Adhering to intention-to-treat principles, none of the enrolled participants were generally excluded. Missing values were replaced by multivariate imputation by chained equations (MICE; with n=5 imputations [51]) based on the control arm, using the variables age and gender as predictors in addition to the T1 outcome measurements. Four additional sensitivity analyses are reported in the online support material (OSM) in Multimedia Appendix 2 (OSM 1-4) for the primary outcomes: A completer analysis using only patient data with completed T1 and T3 measures, last-observation-carried-forward (LOCF), baseline-observation-carried-forward (BOCF), and a reference-based-multiple jump to reference imputation (J2R; [52]). In addition to these analyses, a “per-protocol” sample sensitivity analysis was defined for exploratory analyses, including all IG patients who completed at least 4 of the 12 modules.

The confirmatory analysis of the primary endpoints consisted of calculating a mixed model with 2 measurement time points and a general correlation structure [47]. A random effect for the participant was calculated (random intercept), and 3 fixed effects (group, time, and the interaction of these 2 effects). The 2 measurement times were nested within participants. The treatment effect was estimated as the fixed interaction effect after the final (T3) assessment. To assess the magnitude of the treatment effects, the fixed interaction effect of time and group was divided by the root of the summed variances of the random effects [53]. Effect sizes can be roughly interpreted according to Cohen *d*: effect sizes of 0.20 are considered small, 0.50 moderate, and 0.80 large [54].

Secondary confirmatory outcomes were calculated only after success in the primary analysis to prevent alpha inflation, using the same mixed model with a random intercept for the participant. The CSSRI questionnaire was split into the 3 most relevant subscales (CSSRI-partially inpatient, CSSRI-outpatient, and CSSRI-complementary) that were also subject to Bonferroni-Holm adjustment by dividing by 3, 2, and 1, respectively. Due to highly skewed data, the iPCQ and the CSSRI partially inpatient scales were log_10_-transformed. The CSSRI-outpatient and CSSRI-complementary scales were dichotomized because of rare extreme outliers, in which case a transformation is not recommended [55]. For these dichotomized measures, the analysis was adapted to a mixed logistic regression model to stay as close as possible to the study protocol and odds ratios were calculated as the effect size.

The additional outcome measures, BAI and PHQ-9*,* were analyzed using the same model as the primary and secondary outcomes. Independent *t* tests and chi-square tests were used to estimate differences between groups in pretreatment sample characteristics. Also, *t* tests were used to identify differences in adverse effects in the NEQ at T3.

### Data and Code

All data and analysis code have been made publicly available at the OSF repository and can be accessed at [56]. Materials about the content of the online intervention are reported in the study protocol [29].

### Ethical Considerations

This study was approved by the ethics committee at Heidelberg University in adherence to institutional guidelines (AZ Prüß 2021 1/1). All participants provided informed consent prior to their study participation and were informed that they could withdraw consent anytime. During the study, participant data were pseudonymized by replacing data identifiers (eg, participants’ name) with a pseudonymous code. After study completion, all data were anonymized by deleting the data identifiers. All participants received an allowance of 30€ after completing the questionnaires (T1 and T3). Suicidal thoughts were assessed at multiple time points (T1, T2, and T3) using a Likert scale. If participants rated their thoughts as 1 or higher (indicating they had thoughts of wanting to harm themselves) for the past 2 weeks, they were contacted by phone or email, and an emergency plan was established. For individuals contacted due to suicidality, their questionnaire participation was halted to prioritize immediate support. Suicidal incidents occurred 3 times, all within the CG.

## Results

### Participants Flow

**Figure 1:**
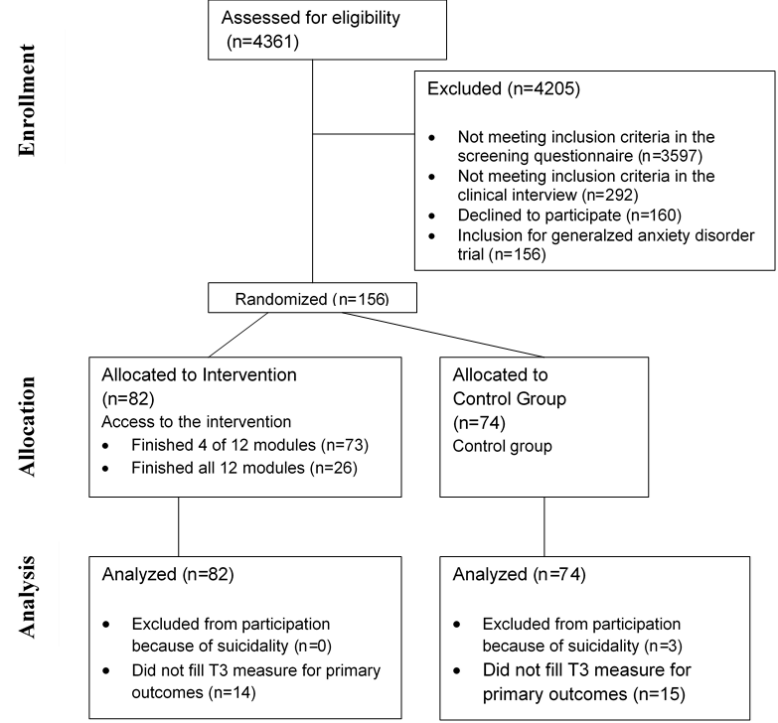
CONSORT flow diagram.

Recruitment occurred from February 12, 2021, to March 21, 2022. A total of 764 clinical interviews were conducted, leading to 292 participants being excluded based on inclusion and exclusion criteria (Figure 1). Further, 160 participants declined study participation and additional 156 participants joined another trial on generalized anxiety disorder [57]. The remaining 156 participants were randomized into the IG (n=82) and CG (n=74). Sociodemographic differences between the groups were not significant.

Deviations from the sample size occurred for some of the secondary and additional outcomes due to partial missingness.

More IG participants (30/81, 35.8%) had a current diagnosis of social phobia compared with CG (11/73, 15.1%, *P*=.005). However, more CG participants had a past diagnosis of social phobia (4/73, 5.5%) than IG (0/81, 0%, *P*=.048). For both current generalized anxiety disorder (31/81, 38.3% vs 17/73, 23.3%, *P*=.06) and current major depression (8/81, 9.8% vs 1/73, 1.4%, *P*=.08) there was a trend toward statistical significance for higher occurrence in the IG. No other diagnosis differences were observed (OSM 6 in Multimedia Appendix 2). Current psychopharmacology (*P=*.81) and psychotherapy (*P=*.34) also did not differ between groups. The CG had lower anxiety levels at baseline for the BAI (*t*_153.8_=2.30, *P*=.02). No group differences were found in primary or secondary outcomes.

### Participants’ Characteristics

The sociodemographic characteristics of all participants are displayed in Table 1. Altogether, out of 194 participants, 59 (38.3%) were diagnosed with panic disorder, whereas 96 (61.7%) fulfilled the diagnostic criteria of panic disorder with additional agoraphobia. Regarding comorbidities, 47 (30.1%) participants also fulfilled the diagnostic criteria for generalized anxiety disorder, which was the most prominent comorbid disorder (OSM 6 in Multimedia Appendix 2). Comorbid depression rates were relatively low, with only 9 (5.8%) participants fulfilling the diagnostic criteria for major depressive disorder. It is important to note that the low comorbidity rate can be attributed to this study’s exclusion criteria, which specifically excluded individuals with severe depression.

**Table 1:** Sociodemographic characteristics of the study cohort at baseline.

Characteristics			Treatment (n=82)		Control (n=74)		Total sample (N=156)
**Sex, n (%)**							
	Female	65 (79.27)		56 (75.68)		121 (77.56)	
	Male	16 (19.51)		17 (22.97)		33 (21.15)	
	Nonbinary	1 (1.22)		1 (1.35)		2 (1.29)	
Age in years (mean, SD)			35.1 (11.5)		35.0 (11.1)		35.0 (11.3)
**Health care use, n (%)**							
	Psychotherapy	35 (42.68)		26 (35.14)		61 (39.10)	
	Pharmacotherapy	27 (32.93)		23 (31.08)		50 (32.05)	
**Relationship status^a^, n (%)**							
	Married	23 (27.71)		26 (35.14)		49 (31.21)	
	Not living with partner	3 (3.61)		0 (0)		3 (1.91)	
	Single	51 (61.45)		45 (60.81)		96 (61.15)	
	Divorced	5 (6.02)		3 (4.05)		8 (5.10)	
	Widowed	1 (1)		0 (0)		1 (0.64)	
**Children, n (%)**							
	Yes	12 (14.63)		19 (25.68)		31 (19.87)	
	No	70 (85.37)		55 (74.32)		125 (80.13)	
**Current diagnoses, n (%)**							
	Panic disorder without agoraphobia	27 (33.3)		32 (43.8)		59 (38.3)	
	Panic disorder with agoraphobia	54 (66.7)		41 (56.2)		96 (61.7)	
	Generalized anxiety disorder	31 (38.3)		17 (23.3)		48 (31.2)	
	Major depressive disorder	8 (9.8)		1 (1.4)		9 (5.8)	
	Social phobia	29 (35.8)		4 (5.5)		40 (26)	

^a^Multiple options were possible.

### Missing Data

Noncompletion rates were 16.0% (25/84) for the PAS (first primary outcome) and 18.6% (29/72) for the WHO-5 (second primary outcome) at T3. Therefore, 70 out of 82 (85.4%) participants in the IG and 61 out of 74 (82.4%) participants in the CG completed the PAS at posttreatment. Similarly, 68 out of 84 (82.3%) participants in the IG and 59 out of 72 (79.7%) participants in the CG completed the WHO-5 post treatment.

Logistic regression analyses showed that participant dropout for the WHO-5 was not associated with any of the following baseline variables: group allocation, sex, age, work capacity, medication intake, or the baseline values of any of the primary or secondary outcomes. For the PAS, dropout was not associated with group allocation, age, work capacity, psychotherapy, or the baseline values of any primary or secondary outcomes. However, female sex (*P*=.02) and medication intake (*P*=.05) were associated with reduced dropout for the PAS.

Since none of the baseline outcomes were associated with dropout, the empirical data supported missing at random instead of missing not at random. Therefore, as determined in the study protocol, MICE analysis was conducted as our primary analysis.

### Adverse Events

During the trial, 3 participants from the CG reported suicidality and had to be excluded from the study. Trained psychologists immediately contacted them. They were excluded from further data collection, but all previous data were still used.

### Adherence

Due to a technical problem, usage data were missing for 1 person in the IG. Based on data from 81 participants, the IG completed an average of 7.3 (SD 3.9) modules out of the total 12 modules, amounting to 7.3 out of 12 (60.8%) of all modules. Overall, 26 out of 84 (32.1%) participants completed the whole course, and 73 out of 84 (90.1%) participants completed the first 4 modules, which was chosen as a sensitivity analysis to assess a basic amount of engagement.

### Primary Outcomes

For the primary outcome PAS (*t*_110.1_*=*–2.22, *P*=.03), we found a significant time×group interaction effect, but not for the primary outcome WHO-5 (*t*_149.8_*=*–1.35, *P*=.09), as shown in Table 2. Effect sizes were small to moderate for the PAS (*d=*–0.37, 95% CI –0.70 to –0.04) and small for the WHO-5 Cohen (*d=*0.22; 95% CI –0.10 to 0.53). Within-group T1-T3 effect sizes for PAS were moderate in the IG Cohen (*d=*–0.58, 95% CI –0.77 to –0.39) and small in the CG Cohen (*d=*–0.21, 95% CI –0.42 to 0.00). Within-group effects were small to moderate in the IG Cohen (*d*=0.30, 95% CI 0.06 to 0.53) and minimal in the CG Cohen (*d=*0.08, 95% CI –0.11 to 0.28) for WHO-5. All imputations and plots can be seen in OSM 1-4 in Multimedia Appendix 2.

**Table 2:** Bonferroni-Holm adjustment for the primary outcomes (multivariate imputation by chained equations imputed).

Primary outcome	*t* test *(df)*	*P* value (1-sided)	Adjustment factor	Adjusted *P* value	Effect size *d* (95% CI)
PAS^a^	–2.22 (110.1)	.01	2	.03	–0.37 (–0.70 to –0.04)
WHO-5^b^	1.35 (149.8)	.09	1	.09	0.22 (–0.10 to 0.53)

^a^PAS: Panic and Agoraphobia Scale.

^b^WHO-5: WHO (World Health Organization)-5 Well-Being Index.

On average, severe levels of panic and agoraphobia were reported at baseline (mean 34.56, SD 8.31; scores range from 13 to 66; Table 3). Moreover, low well-being was reported at baseline (mean 2.87, SD=0.97; scores range from 0.0 to 4.2; Table 3).

**Table 3:** Intention-to-treat data for the Panic and Agoraphobia Scale and the WHO (World Health Organization)-5 Well-Being Index.

Imputation			T1					T2					T3		
			N		Mean (SD)		N			Mean (SD)		N			Mean (SD)
**ITT^a^ PAS^b^**															
	Treatment	82		35.1 (7.93)		66			32.3 (8.41)		70			29.9 (7.90)	
	Control	74		33.9 (8.73)		62			33.7 (7.84)		61			32.7 (8.77)	
**ITT WHO-5^c^**															
	Treatment	82		2.83 (0.94)		64			3.01 (1.01)		68			3.12 (1.06)	
	Control	74		2.92 (1.00)		60			3.03 (1.02)		59			2.99 (1.04)	

^a^ITT: intention-to-treat.

^b^PAS: Panic and Agoraphobia Scale.

^c^WHO-5: WHO (World Health Organization)-5 Well-Being Index.

### Minimal Clinical Important Difference

The Reliable Change Index (RCI; [58]) was used to calculate reliable improvement or deterioration. Regarding the PAS, 33.6% of IG and 16.8% of CG patients improved reliably from T1 to T3. In contrast, 6.1% deteriorated in the IG and 9.2% in the CG. Therefore, a significant difference was found (*P*<.001).

For the WHO-5, improvement (28.3% vs 11.4%) favored the IG. However, deterioration (12.7% vs 11.4%) was stronger in the CG. A significant difference was identified (*P*=.002).

### Sensitivity Analyses for Per-Protocol Sample (4 Completed Modules)

In addition, per-protocol sensitivity analyses were conducted for both primary outcomes, including only the 73 (90.1%) participants who completed at least the first 4 modules. Because no hypotheses were specified, the effects are calculated as 2-tailed tests without alpha adjustment. A significant group×time interaction (**t*_252_=*–3.25, *P=*.001) was found for the PAS with a moderate effect size Cohen (*d=*–0.50; 95% CI –0.80 to –0.20). Regarding the WHO-5, no significant interaction was found (**t*_252_=*1.80, *P=*.07).

### Secondary Outcomes

None of the interaction effects of the secondary outcomes were significant after the Bonferroni-Holm adjustment (Table 4).

On average, impairment of daily functioning (WSAS) was moderate at baseline (mean 4.1, SD 1.7), while mental health literacy (MHLS) levels were very high (mean 4.4, SD 0.4). iPCQ scores and CSSRI outpatient treatment were log-transformed due to the right-skewed distribution (Table 5). Inpatient (CSSRI partly inpatient) and complementary (CSSRI complementary) treatment occurred for 15.3% and 12.1% of participants at baseline (Table 6).

**Table 4:** Linear mixed model and Bonferroni-Holm adjustment for the secondary outcomes based on multiple imputation by chained equations imputation (T1-T3). Effects are odds ratios. The additional factors are due to the additional alpha adjustments of the client sociodemographic and service receipt inventory subscales.

Outcome	Group×time					Effect size *d* (95% CI)
	*t* test *(df)*	*P* value (1-sided)	Adjustment factor	Adjusted *P* value		
WSAS^a^	–.72 (105.1)	.04	4	.17	–0.22 (–0.48 to 0.03)	
MHLS^b^	–0.76 (231.6)	.22	2	.45	–0.09 (–0.34 to 0.15)	
iPCQ^c^	–1.37 (206.8)	.09	3	.26	–0.21 (–0.51 to 0.09)	
CSSRI^d^ outpatient	0.48 (231.6)	1.0	1×1^e^	1.0	0.07 (–0.22 to 0.37)	
CSSRI partly inpatient	–0.56 (6.2)	.29	1×3^e^	.88	0.24^f^ (0 to 30.16)	
CSSRI complementary	–0.30 (6.5)	.39	1×2^e^	.78	0.16^f^ (0 to 28134)	

^a^WSAS: Work and Social Adjustment Scale.

^b^MHLS: Mental Health Literacy Scale.

^c^iPCQ: iMTA Productivity Cost Questionnaire.

^d^CSSRI: Client Sociodemographic and Service Receipt Inventory.

^e^The additional factors are due to the additional α adjustments of the CSSRI subscales.

^f^Effects are odds ratios.

**Table 5:** Intention-to-treat data for the secondary outcomes Work and Social Adjustment Scale, Mental Health Literacy Scale, iMTA Productivity Cost Questionnaire, and client sociodemographic and service receipt inventory outpatient. multiple imputations by chained equations.

Outcome			T1				T2					T3		
			N		Mean (SD)		N		Mean (SD)		N			Mean (SD)
**WSAS^a^**														
	Treatment	82		4.16 (1.67)		82		3.89 (1.72)		82			3.55 (1.68)	
	Control	74		4.12 (1.73)		74		3.87 (1.68)		74			3.90 (1.86)	
**MHLS^b^**														
	Treatment	82		4.38 (0.39)		82		4.37 (0.40)		82			4.35 (0.45)	
	Control	74		4.38 (0.37)		74		4.36 (0.39)		74			4.38 (0.42)	
**iPCQ^c^ log**														
	Treatment	82		0.50 (0.67)		82		0.45 (0.63)		82			0.40 (0.65)	
	Control	74		0.44 (0.64)		74		0.49 (0.68)		74			0.48 (0.74)	
**CSSRI^d^ outpatient log**														
	Treatment	82		1.25 (1.13)		82		1.07 (1.12)		82			0.95 (1.14)	
	Control	74		1.53 (1.06)		74		1.19 (1.09)		74			1.15 (1.11)	

^a^WSAS: Work and Social Adjustment Scale.

^b^MHLS: Mental Health Literacy Scale.

^c^iPCQ: iMTA Productivity Cost Questionnaire.

^d^CSSRI: Client Sociodemographic and Service Receipt Inventory.

**Table 6:** Intention-to-treat data for the secondary outcomes client sociodemographic and service receipt inventory partly inpatient and client sociodemographic and service receipt inventory complementary. multiple imputations by chained equations.

Outcome		T1 cases, n (%)	T2 cases, n (%)	T3 cases, n (%)
**CSSRI^a^ partly inpatient dichotomized**				
	Treatment	82 (16.3)	82 (18.3)	82 (10.7)
	Control	74 (18.6)	74 (14.9)	74 (16.8)
**CSSRI complementary dichotomized**				
	Treatment	82 (15.9)	82 (13.7)	82 (9.3)
	Control	74 (13)	74 (9.5)	74 (9.7)

^a^CSSRI: Client Sociodemographic and Service Receipt Inventory.

### Additional Outcomes

For the BAI, a significant interaction was found at T3 (*t*_206.8_=–4.12, *P*<.001) with a moderate to large effect size Cohen (*d=*–0.60 95% CI –0.89 to –0.32). Within-group effects (T1-T3) were large in the treatment group Cohen (*d=*-0.82 95% CI –1.05 to –0.60) and small in the IG Cohen (*d=*–0.22 95% CI –0.39 to –0.05).

For the PHQ-9, also a significant interaction was found (*t*_257.4_*=–*3.20, *P*<.001) with a small to moderate effect size Cohen (*d=*–0.41 95% CI –0.66 to –0.16). Within-group effects (T1-T3) were small Cohen (*d=*–0.25 95% CI –0.42 to –0.09) in the IG and showed minimal to small deterioration in the CG Cohen (*d=*0.15 95% CI –0.03 to 0.34). Regarding adverse effects (NEQ), no difference was found between the groups (*t*=–1.14, *P*=.26; Table 7 and OSM 5 in Multimedia Appendix 2).

**Table 7:** Most common negative effects as measured by the negative effects questionnaire.

Negative effects	Intervention group (N*=*82), n (%)
Unpleasant memories resurfaced	38 (46.3)
I had more problems with my sleep	15 (18.3)
I felt like I was under more stress	17 (20.7)
I experienced more unpleasant feelings	14 (17.1)
I felt more worried	11 (13.4)
I experienced more anxiety	11 (13.4)
I felt more dejected	10 (12.2)
I stopped thinking help was possible	6 (7.3)
I lost faith in myself	6 (7.3)
I experienced more hopelessness	5 (6.1)

## Discussion

### Principal Findings

This study compared the effectiveness of an online-based self-help iCBT for patients with panic disorder with or without agoraphobia with the care as usual over 12 weeks. In this trial, a significantly greater reduction in panic and agoraphobia symptoms was found for patients using the online intervention. No effects were found regarding the well-being of the patients. Also, no effects were found regarding the secondary hypotheses, namely daily functioning, work capacity, mental health literacy, and health care-related burdens. In the additional outcomes, significantly greater reductions in anxiety symptoms and depression were identified in favor of the online intervention.

In our study, effect sizes for panic and agoraphobia symptoms were moderate within the groups Cohen (*d*=0.58) and small to moderate for the interaction effect Cohen (*d*=0.37). These interaction effects are smaller than the ones reported [21] with *g*=1.04 for panic and *g*=0.64 for agoraphobia when the treatment length was between 5 to 12 weeks. However, a network meta-analysis did only find small Cohen (*d*=.21) and not significant effects for unguided iCBT in comparison to care as usual [27], highlighting a substantial heterogeneity of results.

The small effect sizes in our study may be attributed to several factors. First, even though randomization occurred, the IG was more severely burdened than the CG with higher anxiety levels in the BAI*,* higher rates of social phobia (35.8% vs 15.1%), marginal higher rates of generalized anxiety disorder (38.4% vs 23.3%), and marginal higher rates of depression (9.8% vs 1.4%). These additional burdens mostly concern anxiety symptoms and may have diminished treatment effects in the IG. Second, the study was conducted from March 2021 to February 2022, which corresponds to the COVID-19 pandemic. Preliminary evidence suggests that the severity of panic disorder symptoms increased during the COVID-19 pandemic (Cohen *d=*0.85 during the first wave [59]). This could be plausible, since respiratory difficulties are common symptoms of panic and SARS-CoV-2, possibly leading to breath-related fear conditioning and associated hypervigilance, which could trigger and exacerbate panic symptoms [60] in both the IG and CG. Third, there might have been insufficient treatment adherence in the IG, since on average only 60.8% of the modules were finished and only 32% of participants completed the whole course. Fourth, since treatment effectiveness did not improve in our sensitivity analysis including only patients who had finished at least 4 modules, the intervention may lack effectiveness without guidance. This would not be unexpected, as unguided iCBT for panic disorder was not superior to care as usual in a network meta-analysis [27].

In summary, higher illness burden in the IG as well as insufficient adherence may have diminished the differential effects on panic and agoraphobia symptoms as well as well-being. However, without guidance, the intervention itself may not be sufficient to improve well-being in comparison to care as usual. Simultaneously, the COVID-19 pandemic might have lowered effects in both groups, but cannot explain weakened differential effect sizes.

Regarding the secondary hypotheses, several reasons for the lack of effects come to mind. First, this trial was not specifically powered for the secondary outcomes, especially due to the Bonferroni-Holm adjustment. The minimal effect size for 80% power and α=.0125 was calculated at Cohen *d*=0.46 based on a post-hoc power analysis of the WSAS, so that only moderate to large effects could be detected. Second, the iPCQ and CSSRI subscales had a strong floor effect [61] due to the rare occurrence of additional treatments respective lost working hours at baseline. For the iPCQ, 45.8% (71/155) of participants reported zero lost work hours. For the CSSRI-partly inpatient subscale, 83.3% (120/143) reported no inpatient appointments, and for the CSSRI complementary subscale, 86.7% (124/143) reported no complementary treatments at baseline. Third, as discussed above, insufficient adherence to the intervention may also play a role. Fourth, it must also be considered that the program lacks differential effectiveness without guidance, as has been shown in the literature [27]. In conclusion, the lack of effects on the secondary outcomes could be due to various reasons, but it may also be due to lacking effectiveness without guidance by a therapist.

### Implications for Future Research

In the current literature, most studies focus on panic and agoraphobia symptomatology, and there is little to no research on well-being and functioning. Yet, these measures are essential as they provide a more holistic perspective on recovery [62,63]. Further, as 60% of the financial burden is due to absenteeism [10] and additional costs are resulting from treatment, work capacity, and health care use allow an estimation of the costs associated with panic disorder. This current study found only small and nonsignificant effects on well-being Cohen (*d=*0.22), functioning Cohen (*d*=0.22), and work capacity Cohen (*d*=0.21), which may be due to various reasons (eg, effects do not generalize to more distal outcomes, other problems come to the surface after some symptoms are less severe anymore, lack of guidance diminishes the patients’ ability to follow the program). Therefore, studies with higher statistical power are needed for reliable results. Also, modules specifically addressing these outcomes (eg, well-being and work-related problems) could be integrated into iCBT to broaden the effects. In addition, since insufficient adherence could also explain diminished effects in the IG, it may be worthwhile to edit the intervention to make it more engaging. It might also be necessary to integrate some form of guidance into the intervention to empower patients to follow the program more successfully.

There is some evidence for the cost-effectiveness of panic disorder prevention via face-to-face therapy [64]. Therefore, iCBT seems even more suitable for preventative measures, as it is readily available at disorder onset and cheaper than traditional CBT [20].

Another important line of future research should focus on predictors of treatment effects. Although there are some preliminary findings for moderators (alliance [65] and impairment [66]), further research is warranted to enable clinicians to design more effective treatment courses.

### Limitations

The study had several limitations. First, a care as usual CG was chosen, to reflect routine care in Germany. However, this only allows conclusions in comparison to German routine care and provides no differential effect sizes to other interventions. Second, this study has some deviations from usual care in Germany, which were assessed via the PRECIS-2 (Providing Regional Climates for Impacts Studies) tool ([67]; OSM 7 in Multimedia Appendix 2): Since many patients were recruited via university and social media and not from a mental health provider, this study may only partly reflect routine care. This is also indicated by the finding that our sample was predominantly female odds ratio (OR; OR 6.1), with a substantially higher percentage than in a representative sample from the US population (OR 2.0; [68]). This may not be too surprising since women show generally higher levels of help-seeking [69], but it could also be due to the recruitment strategy, which is a deviation from the usual recruitment. Other deviations include the structured diagnostic interview and the 2 follow-up assessments, which may impede external validity. However, other aspects of the trial (eg, eligibility, delivery, and primary hypotheses) were designed with high external validity, altogether indicating a pragmatic (routine care) trial as assessed via PRECIS-2. Third, this analysis lacked follow-up data, which are essential to test the effects’ durability and assess effects with more extended time frames (eg, CSSRI, iPCQ).

However, this study provides evidence for the effectiveness of the online-based intervention in conditions comparable to routine care. The design allowed for a balance between high internal validity due to computer-generated randomization and blinding of the investigators and high external validity owing to inclusive exclusion criteria that only excluded comorbidities that would affect the use of the intervention while allowing for additional support such as psychotherapy and medication. Regarding the internal validity, randomization was mostly successful with similar levels of concurrent psychotherapy treatment and medication, as well as baseline severity (with the exception of the BAI). Further, this trial investigated vital outcomes such as well-being, everyday functioning, work capacity, and health care burdens, providing valuable insights into the intervention’s impact.

## Appendix

Multimedia Appendix 1 CONSORT e-HEALTH checklist (V 1.6.1).

Multimedia Appendix 2 Online support material.

## Abbreviations

BAI: Beck Anxiety Inventory
BOCF: baseline-observation-carried-forward
CBT: cognitive behavioral therapy
CG: control group
CONSORT-EHEALTH: Consolidated Standards of Reporting Trials of Electronic and Mobile Health Applications and Online Telehealth
CSSRI: Client Sociodemographic and Service Receipt Inventory
DIPS-OA: diagnostisches interview psychischer Störungen – open access
DSM: Diagnostic and Statistical Manual of Mental Disorders
DSM-IV-TR: Diagnostic and Statistical Manual of Mental Disorders (Fourth Edition, Text Revision)
iCBT: internet-based cognitive behavioral therapy
IG: intervention group
iPCQ: iMTA Productivity Cost Questionnaire
J2R: jump to reference imputation
LOCF: last-observation-carried-forward
MHLS: Mental Health Literacy Scale
MICE: multivariate imputation by chained Equations
NEQ: Negative Effects Questionnaire
OR: odds ratio
OSM: online support material
PAS: Panic and Agoraphobia Scale
PHQ-9: Patient Health Questionnaire-9
PRECIS-2: Providing Regional Climates for Impacts Studies
RCI: Reliable Change Index
WSAS: Work and Social Adjustment Scale
WHO-5: World Health Organization–5 Well-Being Index

## Data Availability

The datasets generated and analyzed during this study are available in the OSF Repository [56].
