# Supplementary material for: Evaluation of an Online-Based Self-Help Program for Patients With Panic Disorder: Randomized Controlled Trial
Source: J Med Internet Res. 2025 Apr 2;27:e54062. doi: 10.2196/54062 (PMC12004017; doi:10.2196/54062)
Supplement: Multimedia Appendix 1 [file jmir_v27i1e54062_app1.pdf]

CONSORT-EHEALTH (V 1.6.1) - Submission/Publication Form

The CONSORT-EHEALTH checklist is intended for authors of randomized trials evaluating web-based and Internet-based applications/interventions, including mobile interventions, electronic games (incl multiplayer games), social media, certain telehealth applications, and other interactive and/or networked electronic applications. Some of the items (e.g. all subitems under item 5 - description of the intervention) may also be applicable for other study designs.

The goal of the CONSORT EHEALTH checklist and guideline is to be
a) a guide for reporting for authors of RCTs,
b) to form a basis for appraisal of an ehealth trial (in terms of validity)

CONSORT-EHEALTH items/subitems are MANDATORY reporting items for studies published in the Journal of Medical Internet Research and other journals / scientific societies endorsing the checklist.

Items numbered 1., 2., 3., 4a., 4b etc are original CONSORT or CONSORT-NPT (non-pharmacologic treatment) items.
Items with Roman numerals (i., ii, iii, iv etc.) are CONSORT-EHEALTH extensions/clarifications.

As the CONSORT-EHEALTH checklist is still considered in a formative stage, we would ask that you also RATE ON A SCALE OF 1-5 how important/useful you feel each item is FOR THE PURPOSE OF THE CHECKLIST and reporting guideline (optional).

Mandatory reporting items are marked with a red *.
In the textboxes, either copy & paste the relevant sections from your manuscript into this form - please include any quotes from your manuscript in QUOTATION MARKS,
or answer directly by providing additional information not in the manuscript, or elaborating on why the item was not relevant for this study.

YOUR ANSWERS WILL BE PUBLISHED AS A SUPPLEMENTARY FILE TO YOUR PUBLICATION IN JMIR AND ARE CONSIDERED PART OF YOUR PUBLICATION (IF ACCEPTED).
Please fill in these questions diligently. Information will not be copyedited, so please use proper spelling and grammar, use correct capitalization, and avoid abbreviations.

DO NOT FORGET TO SAVE AS PDF _AND_ CLICK THE SUBMIT BUTTON SO YOUR ANSWERS ARE IN OUR DATABASE !!!

Citation Suggestion (if you append the pdf as Appendix we suggest to cite this paper in the caption):
Eysenbach G, CONSORT-EHEALTH Group
CONSORT-EHEALTH: Improving and Standardizing Evaluation Reports of Web-based and Mobile Health Interventions
J Med Internet Res 2011;13(4):e126

URL: http://www.jmir.org/2011/4/e126/
doi: 10.2196/jmir.1923
PMID: 22209829

108chrisyoga@gmail.com Konto wechseln

Nicht freigegeben

Entwurf gespeichert

* Gibt eine erforderliche Frage an

Your name *

First Last

Christopher Lalk

Primary Affiliation (short), City, Country *

University of Toronto, Toronto, Canada

Osnabrück University, Osnabrück, Germany

Your e-mail address *

abc@gmail.com

christopher.lalk@uni-osnabrueck.de

Title of your manuscript *

Provide the (draft) title of your manuscript.

Evaluation of an Online-Based Self-Help Program for Patients With Panic Disorder - A Randomized Controlled Trial

Name of your App/Software/Intervention *

If there is a short and a long/alternate name, write the short name first and add the long name in brackets.

Selfapy

Evaluated Version (if any)

e.g. "V1", "Release 2017-03-01", "Version 2.0.27913"

54062-833355-3-RV

Language(s) *

What language is the intervention/app in? If multiple languages are available, separate by comma (e.g. "English, French")

German

URL of your Intervention Website or App

e.g. a direct link to the mobile app on app in appstore (itunes, Google Play), or URL of the website. If the intervention is a DVD or hardware, you can also link to an Amazon page.

selfapy.de

URL of an image/screenshot (optional)

Meine Antwort

Accessibility *

Can an enduser access the intervention presently?

- ○ access is free and open
- ○ access only for special usergroups, not open
- ◉ access is open to everyone, but requires payment/subscription/in-app purchases
- ○ app/intervention no longer accessible
- ○ Sonstiges:

Primary Medical Indication/Disease/Condition *

e.g. "Stress", "Diabetes", or define the target group in brackets after the condition, e.g. "Autism (Parents of children with)", "Alzheimers (Informal Caregivers of)"

Panic Disorder

Primary Outcomes measured in trial *

comma-separated list of primary outcomes reported in the trial

Panic and Agoraphobia Scale, World Health Or

Secondary/other outcomes

Are there any other outcomes the intervention is expected to affect?

Work and Social Adjustment Scale, iMTA Productivity Cost Questionnaire, Mental Health Literacy Scale, Client Sociodemographic and Service Receipt Inventory, Negative Effects Questionnaire, Beck Anxiety Inventory, Patient Health Questionnaire-9

Recommended "Dose" *

What do the instructions for users say on how often the app should be used?

- (selected) Approximately Daily
- Approximately Weekly
- Approximately Monthly
- Approximately Yearly
- "as needed"
- Sonstiges:

Approx. Percentage of Users (starters) still using the app as recommended after 3 months *

- unknown / not evaluated
- 0-10%
- 11-20%
- 21-30%
- (selected) 31-40%
- 41-50%
- 51-60%
- 61-70%
- 71%-80%
- 81-90%
- 91-100%
- Sonstiges:

Overall, was the app/intervention effective? *

- ○ yes: all primary outcomes were significantly better in intervention group vs control
- ◉ partly: SOME primary outcomes were significantly better in intervention group vs control
- ○ no statistically significant difference between control and intervention
- ○ potentially harmful: control was significantly better than intervention in one or more outcomes
- ○ inconclusive: more research is needed
- ○ Sonstiges:

Article Preparation Status/Stage *

At which stage in your article preparation are you currently (at the time you fill in this form)

- ○ not submitted yet - in early draft status
- ○ not submitted yet - in late draft status, just before submission
- ○ submitted to a journal but not reviewed yet
- ○ submitted to a journal and after receiving initial reviewer comments
- ◉ submitted to a journal and accepted, but not published yet
- ○ published
- ○ Sonstiges:

Journal *

If you already know where you will submit this paper (or if it is already submitted), please provide the journal name (if it is not JMIR, provide the journal name under "other")

- ◯ not submitted yet / unclear where I will submit this
- ◉ Journal of Medical Internet Research (JMIR)
- ◯ JMIR mHealth and UHealth
- ◯ JMIR Serious Games
- ◯ JMIR Mental Health
- ◯ JMIR Public Health
- ◯ JMIR Formative Research
- ◯ Other JMIR sister journal
- ◯ Sonstiges:

Is this a full powered effectiveness trial or a pilot/feasibility trial? *

- ◯ Pilot/feasibility
- ◉ Fully powered

Manuscript tracking number *

If this is a JMIR submission, please provide the manuscript tracking number under "other" (The ms tracking number can be found in the submission acknowledgement email, or when you login as author in JMIR. If the paper is already published in JMIR, then the ms tracking number is the four-digit number at the end of the DOI, to be found at the bottom of each published article in JMIR)

- ◯ no ms number (yet) / not (yet) submitted to / published in JMIR
- ◉ Sonstiges: 54062

TITLE AND ABSTRACT

1a) TITLE: Identification as a randomized trial in the title

1a) Does your paper address CONSORT item 1a? *

I.e does the title contain the phrase "Randomized Controlled Trial"? (if not, explain the reason under "other")

- (x) yes
- ( ) Sonstiges:

1a-i) Identify the mode of delivery in the title

Identify the mode of delivery. Preferably use “web-based” and/or “mobile” and/or “electronic game” in the title. Avoid ambiguous terms like “online”, “virtual”, “interactive”. Use “Internet-based” only if Intervention includes non-web-based Internet components (e.g. email), use “computer-based” or “electronic” only if offline products are used. Use “virtual” only in the context of “virtual reality” (3-D worlds). Use “online” only in the context of “online support groups”. Complement or substitute product names with broader terms for the class of products (such as “mobile” or “smart phone” instead of “iphone”), especially if the application runs on different platforms.

	1	2	3	4	5	
subitem not at all important	( )	( )	(x)	( )	( )	essential

Auswahl löschen

Does your paper address subitem 1a-i? *

Copy and paste relevant sections from manuscript title (include quotes in quotation marks "like this" to indicate direct quotes from your manuscript), or elaborate on this item by providing additional information not in the ms, or briefly explain why the item is not applicable/relevant for your study

Title: "Evaluation of a Web-Based Self-Help Program for Patients With Panic Disorder - A Randomized Controlled Trial"

1a-ii) Non-web-based components or important co-interventions in title

Mention non-web-based components or important co-interventions in title, if any (e.g., “with telephone support”).

	1	2	3	4	5	
subitem not at all important	○	◉	○	○	○	essential

Auswahl löschen

Does your paper address subitem 1a-ii?

Copy and paste relevant sections from manuscript title (include quotes in quotation marks "like this" to indicate direct quotes from your manuscript), or elaborate on this item by providing additional information not in the ms, or briefly explain why the item is not applicable/relevant for your study

There is no support or guidance, therefore not mentioned.

1a-iii) Primary condition or target group in the title

Mention primary condition or target group in the title, if any (e.g., “for children with Type I Diabetes”) Example: A Web-based and Mobile Intervention with Telephone Support for Children with Type I Diabetes: Randomized Controlled Trial

	1	2	3	4	5	
subitem not at all important	○	○	◉	○	○	essential

Auswahl löschen

Does your paper address subitem 1a-iii? *

Copy and paste relevant sections from manuscript title (include quotes in quotation marks "like this" to indicate direct quotes from your manuscript), or elaborate on this item by providing additional information not in the ms, or briefly explain why the item is not applicable/relevant for your study

Title: "Evaluation of a Web-Based Self-Help Program for Patients With Panic Disorder - A Randomized Controlled Trial"

1b) ABSTRACT: Structured summary of trial design, methods, results, and conclusions

NPT extension: Description of experimental treatment, comparator, care providers, centers, and blinding status.

1b-i) Key features/functionalities/components of the intervention and comparator in the METHODS section of the ABSTRACT

Mention key features/functionalities/components of the intervention and comparator in the abstract. If possible, also mention theories and principles used for designing the site. Keep in mind the needs of systematic reviewers and indexers by including important synonyms. (Note: Only report in the abstract what the main paper is reporting. If this information is missing from the main body of text, consider adding it)

	1	2	3	4	5	
subitem not at all important	○	○	○	◉	○	essential

Auswahl löschen

Does your paper address subitem 1b-i? *

Copy and paste relevant sections from the manuscript abstract (include quotes in quotation marks "like this" to indicate direct quotes from your manuscript), or elaborate on this item by providing additional information not in the ms, or briefly explain why the item is not applicable/relevant for your study

The abstract methods section contains an intervention and control group description ("The intervention group (N=84) received access to a 12-week online self-help program entailing psychoeducation, cognitive restructuring, exposure and mindfulness elements without therapist guidance. The control group (N=72) received care as usual during the study period and was offered the prospect of using the program after 12 weeks").

1b-ii) Level of human involvement in the METHODS section of the ABSTRACT

Clarify the level of human involvement in the abstract, e.g., use phrases like “fully automated” vs. “therapist/nurse/care provider/physician-assisted” (mention number and expertise of providers involved, if any). (Note: Only report in the abstract what the main paper is reporting. If this information is missing from the main body of text, consider adding it)

	1	2	3	4	5	
subitem not at all important	○	○	○	◉	○	essential

Auswahl löschen

Does your paper address subitem 1b-ii?

Copy and paste relevant sections from the manuscript abstract (include quotes in quotation marks "like this" to indicate direct quotes from your manuscript), or elaborate on this item by providing additional information not in the ms, or briefly explain why the item is not applicable/relevant for your study

The abstract methods section contains information on the degree of therapist guidance ("The intervention group (N=84) received access to a 12-week online self-help program entailing psychoeducation, cognitive restructuring, exposure and mindfulness elements without therapist guidance.").

1b-iii) Open vs. closed, web-based (self-assessment) vs. face-to-face assessments in the METHODS section of the ABSTRACT

Mention how participants were recruited (online vs. offline), e.g., from an open access website or from a clinic or a closed online user group (closed usergroup trial), and clarify if this was a purely web-based trial, or there were face-to-face components (as part of the intervention or for assessment). Clearly say if outcomes were self-assessed through questionnaires (as common in web-based trials). Note: In traditional offline trials, an open trial (open-label trial) is a type of clinical trial in which both the researchers and participants know which treatment is being administered. To avoid confusion, use “blinded” or “unblinded” to indicated the level of blinding instead of “open”, as “open” in web-based trials usually refers to “open access” (i.e. participants can self-enrol). (Note: Only report in the abstract what the main paper is reporting. If this information is missing from the main body of text, consider adding it)

	1	2	3	4	5	
subitem not at all important	○	○	○	◉	○	essential

Auswahl löschen

Does your paper address subitem 1b-iii?

Copy and paste relevant sections from the manuscript abstract (include quotes in quotation marks "like this" to indicate direct quotes from your manuscript), or elaborate on this item by providing additional information not in the ms, or briefly explain why the item is not applicable/relevant for your study

"Recruitment was mostly conducted online and the diagnostic interview was conducted by video call while outcome assessment was administered via online questionnaires."

1b-iv) RESULTS section in abstract must contain use data

Report number of participants enrolled/assessed in each group, the use/uptake of the intervention (e.g., attrition/adherence metrics, use over time, number of logins etc.), in addition to primary/secondary outcomes. (Note: Only report in the abstract what the main paper is reporting. If this information is missing from the main body of text, consider adding it)

	1	2	3	4	5	
subitem not at all important	○	○	◉	○	○	essential

Auswahl löschen

Does your paper address subitem 1b-iv?

Copy and paste relevant sections from the manuscript abstract (include quotes in quotation marks "like this" to indicate direct quotes from your manuscript), or elaborate on this item by providing additional information not in the ms, or briefly explain why the item is not applicable/relevant for your study

"The intervention group (N=84) received access to a 12-week online self-help program entailing psychoeducation, cognitive restructuring, exposure and mindfulness elements without therapist guidance. The control group (N=72) received care as usual during the study period and was offered the prospect of using the program after 12 weeks." We did not enter adherence to the abstract to keep the abstract from getting too long as there is a limit of 450 words to the abstract.

1b-v) CONCLUSIONS/DISCUSSION in abstract for negative trials

Conclusions/Discussions in abstract for negative trials: Discuss the primary outcome - if the trial is negative (primary outcome not changed), and the intervention was not used, discuss whether negative results are attributable to lack of uptake and discuss reasons. (Note: Only report in the abstract what the main paper is reporting. If this information is missing from the main body of text, consider adding it)

	1	2	3	4	5	
subitem not at all important	○	○	◉	○	○	essential

Auswahl löschen

Does your paper address subitem 1b-v?

Copy and paste relevant sections from the manuscript abstract (include quotes in quotation marks "like this" to indicate direct quotes from your manuscript), or elaborate on this item by providing additional information not in the ms, or briefly explain why the item is not applicable/relevant for your study

"The presented online intervention can help reduce the core symptomatology of panic disorder and agoraphobia, as well as anxiety symptoms and associated depression. No effects were found for wellbeing and secondary outcomes. This may be due to higher illness burden in the intervention group and possibly the COVID pandemic which caused unique challenges to patients suffering from panic disorder, but could also be due to a lack of adherence." To keep the abstract at a reasonable length, we did not discuss lack of uptake in detail.

INTRODUCTION

2a) In INTRODUCTION: Scientific background and explanation of rationale

2a-i) Problem and the type of system/solution

Describe the problem and the type of system/solution that is object of the study: intended as stand-alone intervention vs. incorporated in broader health care program? Intended for a particular patient population? Goals of the intervention, e.g., being more cost-effective to other interventions, replace or complement other solutions? (Note: Details about the intervention are provided in "Methods" under 5)

	1	2	3	4	5	
subitem not at all important	○	○	○	◉	○	essential

Auswahl löschen

Does your paper address subitem 2a-i? *

Copy and paste relevant sections from the manuscript (include quotes in quotation marks "like this" to indicate direct quotes from your manuscript), or elaborate on this item by providing additional information not in the ms, or briefly explain why the item is not applicable/relevant for your study

The intervention is a stand-alone-intervention for panic disorder. Since results are mixed, we aimed to assess its effectiveness for panic disorder: "Therefore, results regarding the efficacy and effectiveness of unguided iCBT for panic and agoraphobia are mixed, paradoxically indicating similar effects as face-to-face CBT while also indicating no improvement to care as usual."

The intervention should also help with additional outcomes, aiming also at cost-effectiveness: "Also, little research can be found investigating broader effects on well-being, functioning, mental health literacy, working ability, and health care use. These seem particularly relevant to assess effects on cost-effectiveness."

Finally, we aimed to implement the intervention in conditions similar to routine care: "This study aimed to evaluate a 12-week online self-help program (Selfapy) for panic disorder patients with or without agoraphobia within the German healthcare system. For the analysis, we had two objectives: First, we compared the intervention with care as usual, since superiority for unguided self-help towards care as usual has been questioned. Second, we assessed effectiveness for a range of broader outcomes with limited research data (e.g., well-being, functioning)."

2a-ii) Scientific background, rationale: What is known about the (type of) system

Scientific background, rationale: What is known about the (type of) system that is the object of the study (be sure to discuss the use of similar systems for other conditions/diagnoses, if appropiate), motivation for the study, i.e. what are the reasons for and what is the context for this specific study, from which stakeholder viewpoint is the study performed, potential impact of findings [2]. Briefly justify the choice of the comparator.

	1	2	3	4	5	
subitem not at all important	○	○	◉	○	○	essential

Auswahl löschen

Does your paper address subitem 2a-ii? *

Copy and paste relevant sections from the manuscript (include quotes in quotation marks "like this" to indicate direct quotes from your manuscript), or elaborate on this item by providing additional information not in the ms, or briefly explain why the item is not applicable/relevant for your study

There are mixed results for unquided interventions for panic disorder:
"A systematic review and meta-analysis including 27 studies on iCBT for panic disorder [25] showed high efficacy and effectiveness for reducing symptoms of panic disorder (g=1.16) and agoraphobia (g=0.91) compared to waitlist. Another meta-analysis of 13 RCTs for panic disorder and agoraphobia found no efficacy difference between unguided iCBT and face-to-face CBT in terms of panic and agoraphobia symptoms, comorbid depression and anxiety, as well as quality of life improvement [26] indicating that unguided iCBT may be comparably effective to face-to-face CBT. However, in a network meta-analysis of 74 efficacy trials, unguided iCBT was not superior to care as usual for the treatment of panic with or without agoraphobia [27]. In the same analysis, both guided forms of iCBT and face-to-face CBT were superior to care as usual, though not superior to unguided iCBT."

The motivation for the study is: "This study aimed to evaluate a 12-week web-based self-help program (Selfapy) for panic disorder patients with or without agoraphobia within the German healthcare system."

Regarding the comparator: "First, we compared the intervention with care as usual, since superiority for unguided self-help towards care as usual has been questioned."

2b) In INTRODUCTION: Specific objectives or hypotheses

Does your paper address CONSORT subitem 2b? *

Copy and paste relevant sections from the manuscript (include quotes in quotation marks "like this" to indicate direct quotes from your manuscript), or elaborate on this item by providing additional information not in the ms, or briefly explain why the item is not applicable/relevant for your study

"Based on previous research, the primary hypotheses expect superior improvement on panic and agoraphobia symptoms and well-being in the intervention group. The secondary hypotheses expect superior improvement of daily functioning, work capacity, mental health literacy, and the efforts and burdens of patients for the healthcare sector. Lastly, changes in comorbid anxiety and depression symptoms as well as negative effects of the program beyond symptomatology, are being explored as exploratory hypotheses. All outcomes were tested against the control group after 12 weeks."

METHODS

3a) Description of trial design (such as parallel, factorial) including allocation ratio

Does your paper address CONSORT subitem 3a? *

Copy and paste relevant sections from the manuscript (include quotes in quotation marks "like this" to indicate direct quotes from your manuscript), or elaborate on this item by providing additional information not in the ms, or briefly explain why the item is not applicable/relevant for your study

"This parallel group trial was preregistered beforehand [28] and a study protocol was published [29]. Eligible patients were randomly assigned to the intervention group (IG) or control group (CG) in a 1:1 ratio."

3b) Important changes to methods after trial commencement (such as eligibility criteria), with reasons

Does your paper address CONSORT subitem 3b? *

Copy and paste relevant sections from the manuscript (include quotes in quotation marks "like this" to indicate direct quotes from your manuscript), or elaborate on this item by providing additional information not in the ms, or briefly explain why the item is not applicable/relevant for your study

There were changes in the analysis of the outcomes:
"The CSSRI questionnaire was split into the three most relevant subscales (CSSRI-partially inpatient, CSSRI-outpatient, and CSSRI-complementary) that were also subject to Bonferroni-Holm adjustment by dividing by 3, 2, and 1, respectively. Due to highly skewed data, the iPCQ and the CSSRI partially inpatient scales were log10-transformed. The CSSRI-outpatient and CSSRI-complementary scales were dichotomized because of rare extreme outliers, in which case a transformation is not recommended [55]. For these dichotomized measures, the analysis was adapted to a mixed logistic regression model to stay as close as possible to the study protocol and odds ratios were calculated as the effect size."

3b-i) Bug fixes, Downtimes, Content Changes

Bug fixes, Downtimes, Content Changes: ehealth systems are often dynamic systems. A description of changes to methods therefore also includes important changes made on the intervention or comparator during the trial (e.g., major bug fixes or changes in the functionality or content) (5-iii) and other “unexpected events” that may have influenced study design such as staff changes, system failures/downtimes, etc. [2].

	1	2	3	4	5	
subitem not at all important	○	◉	○	○	○	essential

Auswahl löschen

Does your paper address subitem 3b-i?

Copy and paste relevant sections from the manuscript (include quotes in quotation marks "like this" to indicate direct quotes from your manuscript), or elaborate on this item by providing additional information not in the ms, or briefly explain why the item is not applicable/relevant for your study

The intervention stayed the same during the study period. We were not aware of any downtimes, therefore we did not include this section in the manuscript.

4a) Eligibility criteria for participants

Does your paper address CONSORT subitem 4a? *

Copy and paste relevant sections from the manuscript (include quotes in quotation marks "like this" to indicate direct quotes from your manuscript), or elaborate on this item by providing additional information not in the ms, or briefly explain why the item is not applicable/relevant for your study

"Eligible subjects were those who 1) were between 18 and 65 years of age, 2) had sufficient knowledge of the German language, 3) had uninterrupted Internet access and were able to use a computer, 4) provided electronic informed consent to participate in the study, and 5) met criteria for a diagnosis of panic disorder with or without an additional diagnosis of agoraphobia.
Subjects were excluded if they did not meet any of the inclusion criteria or met any of the following criteria: 1) past or current diagnosis of bipolar disorder, 2) past or current diagnosis of psychotic disorder, 3) current diagnosis of substance dependence, 4) current diagnosis of a severe major depressive episode, and 5) acute suicidality. The criteria were chosen, because they could interfere with the successful implementation of the course."

4a-i) Computer / Internet literacy

Computer / Internet literacy is often an implicit “de facto” eligibility criterion - this should be explicitly clarified.

	1	2	3	4	5	
subitem not at all important	○	○	◉	○	○	essential

Auswahl löschen

Does your paper address subitem 4a-i?

Copy and paste relevant sections from the manuscript (include quotes in quotation marks "like this" to indicate direct quotes from your manuscript), or elaborate on this item by providing additional information not in the ms, or briefly explain why the item is not applicable/relevant for your study

"3) had uninterrupted Internet access and were able to use a computer,"

4a-ii) Open vs. closed, web-based vs. face-to-face assessments:

Open vs. closed, web-based vs. face-to-face assessments: Mention how participants were recruited (online vs. offline), e.g., from an open access website or from a clinic, and clarify if this was a purely web-based trial, or there were face-to-face components (as part of the intervention or for assessment), i.e., to what degree got the study team to know the participant. In online-only trials, clarify if participants were quasi-anonymous and whether having multiple identities was possible or whether technical or logistical measures (e.g., cookies, email confirmation, phone calls) were used to detect/prevent these.

	1	2	3	4	5	
subitem not at all important	○	○	●	○	○	essential

Auswahl löschen

Does your paper address subitem 4a-ii? *

Copy and paste relevant sections from the manuscript (include quotes in quotation marks "like this" to indicate direct quotes from your manuscript), or elaborate on this item by providing additional information not in the ms, or briefly explain why the item is not applicable/relevant for your study

"Announcements for study participation were published in the whole of Germany via a university email newsletter, social media, and on flyers in clinics, pharmacies, and practices of medical doctors and psychotherapists. After an online pre-screening, participants were invited to choose an appointment for a remote diagnostic interview assessing the inclusion and exclusion criteria. To this end, a structured diagnostic interview (DIPS-OA, [30-31]) was conducted with every participant via video calls. Participants were not anonymous and multiple identities would likely be detected during the call. "

"Video calls were conducted with all subjects who checked the pre-screening criteria to assess inclusion and exclusion criteria, during which eligibility was assessed via the DIPS-OA. Trained psychologists conducted all interviews under supervision by a certified psychotherapist (CBT)."

4a-iii) Information giving during recruitment

Information given during recruitment. Specify how participants were briefed for recruitment and in the informed consent procedures (e.g., publish the informed consent documentation as appendix, see also item X26), as this information may have an effect on user self-selection, user expectation and may also bias results.

	1	2	3	4	5	
subitem not at all important	○	◉	○	○	○	essential

Auswahl löschen

Does your paper address subitem 4a-iii?

Copy and paste relevant sections from the manuscript (include quotes in quotation marks "like this" to indicate direct quotes from your manuscript), or elaborate on this item by providing additional information not in the ms, or briefly explain why the item is not applicable/relevant for your study

We added the informed consent document to the appendix.

"All participants provided informed consent prior to their study participation and were informed that they could withdraw consent anytime."

4b) Settings and locations where the data were collected

Does your paper address CONSORT subitem 4b? *

Copy and paste relevant sections from the manuscript (include quotes in quotation marks "like this" to indicate direct quotes from your manuscript), or elaborate on this item by providing additional information not in the ms, or briefly explain why the item is not applicable/relevant for your study

The study was conducted online, so people could participate from their homes - recruitment was only conducted in Germany:
"Announcements for study participation were published in the whole of Germany via a university email newsletter, social media, and on flyers in clinics, pharmacies, and practices of medical doctors and psychotherapists."

"The modules contain informative texts, videos, audio, and interactive exercises and can be used via the web as well as on mobile devices from anywhere."

4b-i) Report if outcomes were (self-)assessed through online questionnaires

Clearly report if outcomes were (self-)assessed through online questionnaires (as common in web-based trials) or otherwise.

	1	2	3	4	5	
subitem not at all important	○	○	○	◉	○	essential

Auswahl löschen

Does your paper address subitem 4b-i? *

Copy and paste relevant sections from the manuscript (include quotes in quotation marks "like this" to indicate direct quotes from your manuscript), or elaborate on this item by providing additional information not in the ms, or briefly explain why the item is not applicable/relevant for your study

"Assessment was conducted via an online assessment platform [35].

4b-ii) Report how institutional affiliations are displayed

Report how institutional affiliations are displayed to potential participants [on ehealth media], as affiliations with prestigious hospitals or universities may affect volunteer rates, use, and reactions with regards to an intervention.(Not a required item – describe only if this may bias results)

	1	2	3	4	5	
subitem not at all important	◉	○	○	○	○	essential

Auswahl löschen

Does your paper address subitem 4b-ii?

Copy and paste relevant sections from the manuscript (include quotes in quotation marks "like this" to indicate direct quotes from your manuscript), or elaborate on this item by providing additional information not in the ms, or briefly explain why the item is not applicable/relevant for your study

The study was not conducted at a prestigious institution and we therefore do not think it necessary to discuss this point.

5) The interventions for each group with sufficient details to allow replication, including how and when they were actually administered

5-i) Mention names, credential, affiliations of the developers, sponsors, and owners

Mention names, credential, affiliations of the developers, sponsors, and owners [6] (if authors/evaluators are owners or developer of the software, this needs to be declared in a “Conflict of interest” section or mentioned elsewhere in the manuscript).

	1	2	3	4	5	
subitem not at all important	○	○	○	◉	○	essential

Auswahl löschen

Does your paper address subitem 5-i?

Copy and paste relevant sections from the manuscript (include quotes in quotation marks "like this" to indicate direct quotes from your manuscript), or elaborate on this item by providing additional information not in the ms, or briefly explain why the item is not applicable/relevant for your study

We addressed the issue under "funding": "The study was funded by a European Regional Development Fund awarded to Selfapy. The Professorship for Adult Clinical Psychology and Psychotherapy at Osnabrück University was asked by Selfapy to independently evaluate its product to be assessed to the best of our knowledge and following the standards of good scientific practice. Selfapy made suggestions for the selection of survey instruments in the study's design but had no influence on the data collection, analysis, and interpretation or production of the resulting publications." We were not involved in the creation of the intervention nor do we have any associations with Selfapy.

5-ii) Describe the history/development process

Describe the history/development process of the application and previous formative evaluations (e.g., focus groups, usability testing), as these will have an impact on adoption/use rates and help with interpreting results.

	1	2	3	4	5	
subitem not at all important	(•)	( )	( )	( )	( )	essential

Auswahl löschen

Does your paper address subitem 5-ii?

Copy and paste relevant sections from the manuscript (include quotes in quotation marks "like this" to indicate direct quotes from your manuscript), or elaborate on this item by providing additional information not in the ms, or briefly explain why the item is not applicable/relevant for your study

We did not develop the intervention and did not receive any information from Selfapy regarding the history of the intervention.

5-iii) Revisions and updating

Revisions and updating. Clearly mention the date and/or version number of the application/intervention (and comparator, if applicable) evaluated, or describe whether the intervention underwent major changes during the evaluation process, or whether the development and/or content was “frozen” during the trial. Describe dynamic components such as news feeds or changing content which may have an impact on the replicability of the intervention (for unexpected events see item 3b).

	1	2	3	4	5	
subitem not at all important	( )	(•)	( )	( )	( )	essential

Auswahl löschen

Does your paper address subitem 5-iii?

Copy and paste relevant sections from the manuscript (include quotes in quotation marks "like this" to indicate direct quotes from your manuscript), or elaborate on this item by providing additional information not in the ms, or briefly explain why the item is not applicable/relevant for your study

"The intervention stayed frozen throughout the trial to ensure that all participants underwent exactly the same intervention."

5-iv) Quality assurance methods

Provide information on quality assurance methods to ensure accuracy and quality of information provided [1], if applicable.

	1	2	3	4	5	
subitem not at all important	○	◉	○	○	○	essential

Auswahl löschen

Does your paper address subitem 5-iv?

Copy and paste relevant sections from the manuscript (include quotes in quotation marks "like this" to indicate direct quotes from your manuscript), or elaborate on this item by providing additional information not in the ms, or briefly explain why the item is not applicable/relevant for your study

Unfortunately we did not receive information from Selfapy regarding quality assurance. However, since Selfapy is a medical product in the European Union, it must adhere to strict quality standards.

5-v) Ensure replicability by publishing the source code, and/or providing screenshots/screen-capture video, and/or providing flowcharts of the algorithms used

Ensure replicability by publishing the source code, and/or providing screenshots/screen-capture video, and/or providing flowcharts of the algorithms used. Replicability (i.e., other researchers should in principle be able to replicate the study) is a hallmark of scientific reporting.

	1	2	3	4	5	
subitem not at all important	○	○	○	◉	○	essential

Auswahl löschen

Does your paper address subitem 5-v?

Copy and paste relevant sections from the manuscript (include quotes in quotation marks "like this" to indicate direct quotes from your manuscript), or elaborate on this item by providing additional information not in the ms, or briefly explain why the item is not applicable/relevant for your study

"All data and analysis code have been made publicly available at the OSF repository and can be accessed at [56]. Materials about the content of the online intervention are reported in the study protocol [29]."

5-vi) Digital preservation

Digital preservation: Provide the URL of the application, but as the intervention is likely to change or disappear over the course of the years; also make sure the intervention is archived (Internet Archive, webcitation.org, and/or publishing the source code or screenshots/videos alongside the article). As pages behind login screens cannot be archived, consider creating demo pages which are accessible without login.

	1	2	3	4	5	
subitem not at all important	○	◉	○	○	○	essential

Auswahl löschen

Does your paper address subitem 5-vi?

Copy and paste relevant sections from the manuscript (include quotes in quotation marks "like this" to indicate direct quotes from your manuscript), or elaborate on this item by providing additional information not in the ms, or briefly explain why the item is not applicable/relevant for your study

Since the study is already finished and was finished in 2021, this is unfortunately not possible anymore.

5-vii) Access

Access: Describe how participants accessed the application, in what setting/context, if they had to pay (or were paid) or not, whether they had to be a member of specific group. If known, describe how participants obtained “access to the platform and Internet” [1]. To ensure access for editors/reviewers/readers, consider to provide a “backdoor” login account or demo mode for reviewers/readers to explore the application (also important for archiving purposes, see vi).

	1	2	3	4	5	
subitem not at all important	○	◉	○	○	○	essential

Auswahl löschen

Does your paper address subitem 5-vii? *

Copy and paste relevant sections from the manuscript (include quotes in quotation marks "like this" to indicate direct quotes from your manuscript), or elaborate on this item by providing additional information not in the ms, or briefly explain why the item is not applicable/relevant for your study

"The modules contain informative texts, videos, audio, and interactive exercises and can be used via the web as well as on mobile devices from anywhere".

5-viii) Mode of delivery, features/functionalities/components of the intervention and comparator, and the theoretical framework

Describe mode of delivery, features/functionalities/components of the intervention and comparator, and the theoretical framework [6] used to design them (instructional strategy [1], behaviour change techniques, persuasive features, etc., see e.g., [7, 8] for terminology). This includes an in-depth description of the content (including where it is coming from and who developed it) [1]," whether [and how] it is tailored to individual circumstances and allows users to track their progress and receive feedback" [6]. This also includes a description of communication delivery channels and – if computer-mediated communication is a component – whether communication was synchronous or asynchronous [6]. It also includes information on presentation strategies [1], including page design principles, average amount of text on pages, presence of hyperlinks to other resources, etc. [1].

	1	2	3	4	5	
subitem not at all important	○	○	○	◉	○	essential

Auswahl löschen

Does your paper address subitem 5-viii? *

Copy and paste relevant sections from the manuscript (include quotes in quotation marks "like this" to indicate direct quotes from your manuscript), or elaborate on this item by providing additional information not in the ms, or briefly explain why the item is not applicable/relevant for your study

"The online self-help program for the treatment of panic disorder with or without agoraphobia (Selfapy) was conducted as the intervention. The program is based on evidence-based methods and exercises derived from CBT (such as psychoeducation, cognitive restructuring or exposure), as well as elements from Mindfulness-Based Therapy (e.g., [33-34]). The online intervention consists of core modules, which include mandatory exercise content, and a subsequent set of optional specialization areas that are individually selectable (for a complete overview, see [29]). Each module covers a specific topic, such as exposure, mindfulness, or problem-solving training. The modules contain informative texts, videos, audio, and interactive exercises and can be used via the web as well as on mobile devices from anywhere. The intervention was not changed throughout the trial to ensure that all participants underwent exactly the same intervention.
Participants completed the online program independently and without support. They were monitored for suicidality, in which case they were messaged by a psychologist. Active communication only occurred for safety reasons. The intervention group had immediate access to the 12-week self-help treatment and were advised to spend 15-20 minutes daily on it."
We provided a comprehensive overview in the study protocol, which we referenced in the text and which is publically available.

5-ix) Describe use parameters

Describe use parameters (e.g., intended "doses" and optimal timing for use). Clarify what instructions or recommendations were given to the user, e.g., regarding timing, frequency, heaviness of use, if any, or was the intervention used ad libitum.

	1	2	3	4	5	
subitem not at all important	○	○	○	◉	○	essential

Auswahl löschen

Does your paper address subitem 5-ix?

Copy and paste relevant sections from the manuscript (include quotes in quotation marks "like this" to indicate direct quotes from your manuscript), or elaborate on this item by providing additional information not in the ms, or briefly explain why the item is not applicable/relevant for your study

"The intervention group had immediate access to the 12-week self-help treatment and were advised to spend 15-20 minutes daily on it."

5-x) Clarify the level of human involvement

Clarify the level of human involvement (care providers or health professionals, also technical assistance) in the e-intervention or as co-intervention (detail number and expertise of professionals involved, if any, as well as "type of assistance offered, the timing and frequency of the support, how it is initiated, and the medium by which the assistance is delivered". It may be necessary to distinguish between the level of human involvement required for the trial, and the level of human involvement required for a routine application outside of a RCT setting (discuss under item 21 – generalizability).

	1	2	3	4	5	
subitem not at all important	○	○	○	◉	○	essential

Auswahl löschen

Does your paper address subitem 5-x?

Copy and paste relevant sections from the manuscript (include quotes in quotation marks "like this" to indicate direct quotes from your manuscript), or elaborate on this item by providing additional information not in the ms, or briefly explain why the item is not applicable/relevant for your study

"Participants completed the online program independently and without support. They were monitored for suicidality, in which case they were messaged by a psychologist. Active communication only occurred for safety reasons."

5-xi) Report any prompts/reminders used

Report any prompts/reminders used: Clarify if there were prompts (letters, emails, phone calls, SMS) to use the application, what triggered them, frequency etc. It may be necessary to distinguish between the level of prompts/reminders required for the trial, and the level of prompts/reminders for a routine application outside of a RCT setting (discuss under item 21 – generalizability).

	1	2	3	4	5	
subitem not at all important	○	○	○	◉	○	essential

Auswahl löschen

Does your paper address subitem 5-xi? *

Copy and paste relevant sections from the manuscript (include quotes in quotation marks "like this" to indicate direct quotes from your manuscript), or elaborate on this item by providing additional information not in the ms, or briefly explain why the item is not applicable/relevant for your study

There were no prompts or reminders used for the application of the intervention, which does not change in the context of routine care.

5-xii) Describe any co-interventions (incl. training/support)

Describe any co-interventions (incl. training/support): Clearly state any interventions that are provided in addition to the targeted eHealth intervention, as ehealth intervention may not be designed as stand-alone intervention. This includes training sessions and support [1]. It may be necessary to distinguish between the level of training required for the trial, and the level of training for a routine application outside of a RCT setting (discuss under item 21 – generalizability.

	1	2	3	4	5	
subitem not at all important	○	○	○	◉	○	essential

Auswahl löschen

Does your paper address subitem 5-xii? *

Copy and paste relevant sections from the manuscript (include quotes in quotation marks "like this" to indicate direct quotes from your manuscript), or elaborate on this item by providing additional information not in the ms, or briefly explain why the item is not applicable/relevant for your study

There were no co-interventions and no training or support, which does also not change in routine care.

6a) Completely defined pre-specified primary and secondary outcome measures, including how and when they were assessed

Does your paper address CONSORT subitem 6a? *

Copy and paste relevant sections from the manuscript (include quotes in quotation marks "like this" to indicate direct quotes from your manuscript), or elaborate on this item by providing additional information not in the ms, or briefly explain why the item is not applicable/relevant for your study

"Interim and final evaluations occurred 6 (T2) and 12 (T3) weeks after the baseline assessment (T1)."
"Measures were conducted at three different points in time: Before the start of the intervention (T1, baseline), after six weeks (T2, during treatment), and 12 weeks after the beginning of the intervention (T3, posttreatment). At each measurement time point the primary, secondary and exploratory outcomes were assessed. Assessment was conducted via an online assessment platform [35]."
"The change in panic and agoraphobia symptoms was evaluated using the Panic and Agoraphobia Scale (PAS; [36]). The PAS consists of 13 items that are rated on a five-point Likert scale. There scale contains five subscales: panic attacks, agoraphobic avoidance, anticipatory avoidance, disability, and worries about health. In our data, we calculated McDonald's omega [37] as a reliability measure with ω=0.86 at T1. Wellbeing was assessed by the five-item World Health Organization Well-being Index (WHO-5; [38]). The WHO-5 contains items measuring positive mood, calmness, high energy levels, good rest, and interest in daily activities."
"Functioning in daily life was measured by the Work and Social Adjustment Scale (WSAS; [39) assessing professional and personal functioning. Reliability was good with ω=0.79 at T1.
Work capacity was measured with the iMTA Productivity Cost Questionnaire to assess the amount of lost working hours in the last four weeks due to absenteeism or distress-related impaired work capabilities (iPCQ; [40]).
Mental health literacy was measured with the Mental Health Literacy Scale (MHLS; [41]) with high reliability (ω=0.85 at T1).
The extent of therapy-related efforts and burdens of patients (Client Sociodemographic and Service Receipt Inventory - CSSRI; [42]) was collected on three subscales: CSSRI-partly inpatient to assess partly inpatient treatment, CSSRI-complementary to assess complementary services (e.g., self-help groups), and CSSRI-ambulant to assess outpatient services (e.g., psychotherapy treatment, medical treatment)."
"Adverse treatment effects were assessed with the Negative Effects Questionnaire (NEQ; [43]). The NEQ contains 32 items and showed high reliability (ω=0.89 at T3). Also, general symptoms of anxiety were assessed using the Beck Anxiety Inventory (BAI; [44]) with ω=0.89 at T1. Depressive symptoms were collected with the Patient Health Questionnaire-9 (PHQ-9; [45]) with ω=0.74 at T1."

6a-i) Online questionnaires: describe if they were validated for online use and apply CHERRIES items to describe how the questionnaires were designed/deployed

If outcomes were obtained through online questionnaires, describe if they were validated for online use and apply CHERRIES items to describe how the questionnaires were designed/deployed [9].

	1	2	3	4	5	
subitem not at all important	(x)	( )	( )	( )	( )	essential

Auswahl löschen

Does your paper address subitem 6a-i?

Copy and paste relevant sections from manuscript text

The paper does not address this issue, however, most questionnaires are extremely common and will likely have been used in this context already.

6a-ii) Describe whether and how “use” (including intensity of use/dosage) was defined/measured/monitored

Describe whether and how “use” (including intensity of use/dosage) was defined/measured/monitored (logins, logfile analysis, etc.). Use/adoption metrics are important process outcomes that should be reported in any ehealth trial.

	1	2	3	4	5	
subitem not at all important	( )	( )	(x)	( )	( )	essential

Auswahl löschen

Does your paper address subitem 6a-ii?

Copy and paste relevant sections from manuscript text

"As a measure of adherence the amount of completed modules was tracked, which could entail 12 modules in total."

6a-iii) Describe whether, how, and when qualitative feedback from participants was obtained

Describe whether, how, and when qualitative feedback from participants was obtained (e.g., through emails, feedback forms, interviews, focus groups).

	1	2	3	4	5	
subitem not at all important	(x)	( )	( )	( )	( )	essential

Auswahl löschen

Does your paper address subitem 6a-iii?

Copy and paste relevant sections from manuscript text

No qualitative feedback was obtained from patients.

6b) Any changes to trial outcomes after the trial commenced, with reasons

Does your paper address CONSORT subitem 6b? *

Copy and paste relevant sections from the manuscript (include quotes in quotation marks "like this" to indicate direct quotes from your manuscript), or elaborate on this item by providing additional information not in the ms, or briefly explain why the item is not applicable/relevant for your study

"The CSSRI questionnaire was split into the three most relevant subscales (CSSRI-partially inpatient, CSSRI-outpatient, and CSSRI-complementary) that were also subject to Bonferroni-Holm adjustment by dividing by 3, 2, and 1, respectively. Due to highly skewed data, the iPCQ and the CSSRI partially inpatient scales were log10-transformed. The CSSRI-outpatient and CSSRI-complementary scales were dichotomized because of rare extreme outliers, in which case a transformation is not recommended [55]. For these dichotomized measures, the analysis was adapted to a mixed logistic regression model to stay as close as possible to the study protocol and odds ratios were calculated as the effect size."

Due to not fulfilling the distribution criteria for our mixed model, we log10-transformed two outcomes and dichotomized two other outcomes as mentioned above.

7a) How sample size was determined

NPT: When applicable, details of whether and how the clustering by care provides or centers was addressed

7a-i) Describe whether and how expected attrition was taken into account when calculating the sample size

Describe whether and how expected attrition was taken into account when calculating the sample size.

	1	2	3	4	5	
subitem not at all important	○	○	○	◉	○	essential

Auswahl löschen

Does your paper address subitem 7a-i?

Copy and paste relevant sections from manuscript title (include quotes in quotation marks "like this" to indicate direct quotes from your manuscript), or elaborate on this item by providing additional information not in the ms, or briefly explain why the item is not applicable/relevant for your study

"The between-group effect size estimate was based on meta-analytic evidence for effect sizes in unguided online psychological interventions for anxiety disorders (d=0.45; e.g., [46]). This effect was used as the basis for sample size determination. For the planned mixed model with two measurement time points with a general correlation structure [47], a directed hypothesis, a group allocation of 1:1, a power of 0.80, and an alpha level of 0.025 after Bonferroni-Holm correction, a total of 156 patients (78 per group) were needed. The number of cases was calculated using the R-tool longpower [48]. For the secondary outcomes, we calculated a minimal detectable effect size of d=-0.46 with 80% power and an alpha level of 0.0125 (Bonferroni-Holm adjustment) based on a post-hoc power analysis of the WSAS with simr [49]."

7b) When applicable, explanation of any interim analyses and stopping guidelines

Does your paper address CONSORT subitem 7b? *

Copy and paste relevant sections from the manuscript (include quotes in quotation marks "like this" to indicate direct quotes from your manuscript), or elaborate on this item by providing additional information not in the ms, or briefly explain why the item is not applicable/relevant for your study

There were no interim analyses or stopping guidelines.

8a) Method used to generate the random allocation sequence

NPT: When applicable, how care providers were allocated to each trial group

Does your paper address CONSORT subitem 8a? *

Copy and paste relevant sections from the manuscript (include quotes in quotation marks "like this" to indicate direct quotes from your manuscript), or elaborate on this item by providing additional information not in the ms, or briefly explain why the item is not applicable/relevant for your study

"Randomization in 1:1 ratio without stratification or blocks was done automatically by a computer-generated code. Participants were automatically informed of their group via email. Data collection, evaluation, and statistical analysis were done blindly. A team member not involved in the analysis coded the group variable, and analysis scripts were prepared before knowing the actual data."

8b) Type of randomisation; details of any restriction (such as blocking and block size)

Does your paper address CONSORT subitem 8b? *

Copy and paste relevant sections from the manuscript (include quotes in quotation marks "like this" to indicate direct quotes from your manuscript), or elaborate on this item by providing additional information not in the ms, or briefly explain why the item is not applicable/relevant for your study

There were no blocks: "Randomization in 1:1 ratio without stratification or blocks was done automatically by a computer-generated code."

9) Mechanism used to implement the random allocation sequence (such as sequentially numbered containers), describing any steps taken to conceal the sequence until interventions were assigned

Does your paper address CONSORT subitem 9? *

Copy and paste relevant sections from the manuscript (include quotes in quotation marks "like this" to indicate direct quotes from your manuscript), or elaborate on this item by providing additional information not in the ms, or briefly explain why the item is not applicable/relevant for your study

"Randomization in 1:1 ratio without stratification or blocks was done automatically by a computer-generated code. Participants were automatically informed of their group via email. Data collection, evaluation, and statistical analysis were done blindly. A team member not involved in the analysis coded the group variable, and analysis scripts were prepared before knowing the actual data." The codes were concealed because they were sent automatically via email and were managed by a team member not involved in the analysis.

10) Who generated the random allocation sequence, who enrolled participants, and who assigned participants to interventions

Does your paper address CONSORT subitem 10? *

Copy and paste relevant sections from the manuscript (include quotes in quotation marks "like this" to indicate direct quotes from your manuscript), or elaborate on this item by providing additional information not in the ms, or briefly explain why the item is not applicable/relevant for your study

"Participants were automatically informed of their group via email.. Data collection, evaluation, and statistical analysis were done blindly. A team member not involved in the analysis coded the group variable, and analysis scripts were prepared before knowing the actual data."

11a) If done, who was blinded after assignment to interventions (for example, participants, care providers, those assessing outcomes) and how

NPT: Whether or not administering co-interventions were blinded to group assignment

11a-i) Specify who was blinded, and who wasn't

Specify who was blinded, and who wasn't. Usually, in web-based trials it is not possible to blind the participants [1, 3] (this should be clearly acknowledged), but it may be possible to blind outcome assessors, those doing data analysis or those administering co-interventions (if any).

	1	2	3	4	5	
subitem not at all important	○	○	○	◉	○	essential

Auswahl löschen

Does your paper address subitem 11a-i? *

Copy and paste relevant sections from the manuscript (include quotes in quotation marks "like this" to indicate direct quotes from your manuscript), or elaborate on this item by providing additional information not in the ms, or briefly explain why the item is not applicable/relevant for your study

Patients were not blinded since this was not possible. But the outcome analysis was conducted blindly: "Data collection, evaluation, and statistical analysis were done blindly. A team member not involved in the analysis coded the group variable, and analysis scripts were prepared before knowing the actual data."

11a-ii) Discuss e.g., whether participants knew which intervention was the "intervention of interest" and which one was the "comparator"

Informed consent procedures (4a-ii) can create biases and certain expectations - discuss e.g., whether participants knew which intervention was the "intervention of interest" and which one was the "comparator".

	1	2	3	4	5	
subitem not at all important	○	○	○	◉	○	essential

Auswahl löschen

Does your paper address subitem 11a-ii?

Copy and paste relevant sections from the manuscript (include quotes in quotation marks "like this" to indicate direct quotes from your manuscript), or elaborate on this item by providing additional information not in the ms, or briefly explain why the item is not applicable/relevant for your study

"Since the participants had to actively access the intervention, they were not blinded, as this is generally not possible in psychotherapeutic trials."

11b) If relevant, description of the similarity of interventions

(this item is usually not relevant for ehealth trials as it refers to similarity of a placebo or sham intervention to a active medication/intervention)

Does your paper address CONSORT subitem 11b? *

Copy and paste relevant sections from the manuscript (include quotes in quotation marks "like this" to indicate direct quotes from your manuscript), or elaborate on this item by providing additional information not in the ms, or briefly explain why the item is not applicable/relevant for your study

There were no different interventions.

12a) Statistical methods used to compare groups for primary and secondary outcomes

NPT: When applicable, details of whether and how the clustering by care providers or centers was addressed

Does your paper address CONSORT subitem 12a? *

Copy and paste relevant sections from the manuscript (include quotes in quotation marks "like this" to indicate direct quotes from your manuscript), or elaborate on this item by providing additional information not in the ms, or briefly explain why the item is not applicable/relevant for your study

"The confirmatory analysis of the primary endpoints consisted of calculating a mixed model with two measurement time points and a general correlation structure [47]. A random effect for the subject was calculated (random intercept), and three fixed effects (group, time, and the interaction of these two effects). The two measurement times were nested within subjects. The treatment effect was estimated as the fixed interaction effect after the final (T3) assessment. To assess the magnitude of the treatment effects, the fixed interaction effect of time and group was divided by the root of the summed variances of the random effects [53]. Effect sizes can be roughly interpreted according to Cohen's d: Effect sizes of 0.20 are considered small, 0.50 moderate, and 0.80 large [54].
Secondary confirmatory outcomes were calculated only after success in the primary analysis to prevent alpha inflation, using the same mixed model with a random intercept for the subject."

12a-i) Imputation techniques to deal with attrition / missing values

Imputation techniques to deal with attrition / missing values: Not all participants will use the intervention/comparator as intended and attrition is typically high in ehealth trials. Specify how participants who did not use the application or dropped out from the trial were treated in the statistical analysis (a complete case analysis is strongly discouraged, and simple imputation techniques such as LOCF may also be problematic [4]).

	1	2	3	4	5	
subitem not at all important	○	○	○	●	○	essential

Auswahl löschen

Does your paper address subitem 12a-i? *

Copy and paste relevant sections from the manuscript (include quotes in quotation marks "like this" to indicate direct quotes from your manuscript), or elaborate on this item by providing additional information not in the ms, or briefly explain why the item is not applicable/relevant for your study

"Adhering to ITT principles, none of the enrolled participants were generally excluded. Missing values were replaced by Multivariate Imputation by Chained Equations (MICE; with n=5 imputations; [51]) based on the control arm, using the variables age and gender as predictors in addition to the T1 outcome measurements. Four additional sensitivity analyses are reported in the OSM for the primary outcomes: A completer analysis using only patient data with completed T1 and T3 measures, last-observation-carried-forward (LOCF), baseline-observation-carried-forward (BOCF), and a reference-based-multiple imputation (J2R; [52])."

12b) Methods for additional analyses, such as subgroup analyses and adjusted analyses

Does your paper address CONSORT subitem 12b? *

Copy and paste relevant sections from the manuscript (include quotes in quotation marks "like this" to indicate direct quotes from your manuscript), or elaborate on this item by providing additional information not in the ms, or briefly explain why the item is not applicable/relevant for your study

"In addition to these analyses, a "per-protocol" sample sensitivity analysis was defined for exploratory analyses, including all IG patients who completed at least 4 of the 12 modules."

"The additional outcome measures, BAI and PHQ-9, were analyzed using the same model as the primary and secondary outcomes. Independent t-tests and χ^2-tests were used to estimate differences between groups in pre-treatment sample characteristics. Also, t-tests were used to identify differences in adverse effects in the NEQ at T3."

X26) REB/IRB Approval and Ethical Considerations [recommended as subheading under "Methods"] (not a CONSORT item)

X26-i) Comment on ethics committee approval

	1	2	3	4	5	
subitem not at all important	○	○	○	◉	○	essential

Auswahl löschen

Does your paper address subitem X26-i?

Copy and paste relevant sections from the manuscript (include quotes in quotation marks "like this" to indicate direct quotes from your manuscript), or elaborate on this item by providing additional information not in the ms, or briefly explain why the item is not applicable/relevant for your study

"This study was approved by the ethics committee at Heidelberg University in adherence to institutional guidelines (Ethics Committee-No. AZ Prüß 2021 1/1)."

x26-ii) Outline informed consent procedures

Outline informed consent procedures e.g., if consent was obtained offline or online (how? Checkbox, etc.?), and what information was provided (see 4a-ii). See [6] for some items to be included in informed consent documents.

	1	2	3	4	5	
subitem not at all important	○	○	◉	○	○	essential

Auswahl löschen

Does your paper address subitem X26-ii?

Copy and paste relevant sections from the manuscript (include quotes in quotation marks "like this" to indicate direct quotes from your manuscript), or elaborate on this item by providing additional information not in the ms, or briefly explain why the item is not applicable/relevant for your study

All participants provided informed consent prior to their study participation and were informed that they could withdraw consent anytime." This was conducted online, but was also discussed during the clinical interview.

X26-iii) Safety and security procedures

Safety and security procedures, incl. privacy considerations, and any steps taken to reduce the likelihood or detection of harm (e.g., education and training, availability of a hotline)

	1	2	3	4	5	
subitem not at all important	○	○	◉	○	○	essential

Auswahl löschen

Does your paper address subitem X26-iii?

Copy and paste relevant sections from the manuscript (include quotes in quotation marks "like this" to indicate direct quotes from your manuscript), or elaborate on this item by providing additional information not in the ms, or briefly explain why the item is not applicable/relevant for your study

"Participants completed the online program independently and without support. They were monitored for suicidality, in which case they were messaged by a psychologist. Active communication only occurred for safety reasons."

"Suicidal thoughts were assessed at multiple time points (T1, T2, and T3) using a Likert scale. If participants rated their thoughts as 1 or higher (indicating they had thoughts of wanting to harm themselves) for the past two weeks, they were contacted by phone or email, and an emergency plan was established. For individuals contacted due to suicidality, their questionnaire participation was halted to prioritize immediate support. Suicidal incidents occurred three times, all within the control group."

RESULTS

13a) For each group, the numbers of participants who were randomly assigned, received intended treatment, and were analysed for the primary outcome

NPT: The number of care providers or centers performing the intervention in each group and the number of patients treated by each care provider in each center

Does your paper address CONSORT subitem 13a? *

Copy and paste relevant sections from the manuscript (include quotes in quotation marks "like this" to indicate direct quotes from your manuscript), or elaborate on this item by providing additional information not in the ms, or briefly explain why the item is not applicable/relevant for your study

"The remaining 156 participants were randomized into the Intervention Group (IG, N=84) and Control Group (CG, N=74)." They were included into the intention-to-treat-Analysis. There were no different centers, since participation was done online.

13b) For each group, losses and exclusions after randomisation, together with reasons

Does your paper address CONSORT subitem 13b? (NOTE: Preferably, this is shown in a CONSORT flow diagram) *

Copy and paste relevant sections from the manuscript (include quotes in quotation marks "like this" to indicate direct quotes from your manuscript), or elaborate on this item by providing additional information not in the ms, or briefly explain why the item is not applicable/relevant for your study

CONSORT diagram shows this in the manuscript.

13b-i) Attrition diagram

Strongly recommended: An attrition diagram (e.g., proportion of participants still logging in or using the intervention/comparator in each group plotted over time, similar to a survival curve) or other figures or tables demonstrating usage/dose/engagement.

	1	2	3	4	5	
subitem not at all important	○	○	◉	○	○	essential

Auswahl löschen

Does your paper address subitem 13b-i?

Copy and paste relevant sections from the manuscript or cite the figure number if applicable (include quotes in quotation marks "like this" to indicate direct quotes from your manuscript), or elaborate on this item by providing additional information not in the ms, or briefly explain why the item is not applicable/relevant for your study

"Due to a technical problem, usage data was missing for one person in the IG. Based on data from 81 participants, the IG completed an average of 7.3 (sd=3.9) modules out of the total 12 modules, amounting to 60.8% of all modules. 26 (32.1%) participants completed the whole course, and 73 (90.1%) underwent the first four modules, which was chosen as a sensitivity analysis to assess a basic amount of engagement." We did not provide an attrition diagram, but described adherence in the text.

14a) Dates defining the periods of recruitment and follow-up

Does your paper address CONSORT subitem 14a? *

Copy and paste relevant sections from the manuscript (include quotes in quotation marks "like this" to indicate direct quotes from your manuscript), or elaborate on this item by providing additional information not in the ms, or briefly explain why the item is not applicable/relevant for your study

"Recruitment occurred from February 12, 2021, to March 21, 2022. A total of 764 clinical interviews were conducted, leading to 292 participants being excluded based on inclusion and exclusion criteria (see Figure 1)." There was no follow-up.

14a-i) Indicate if critical “secular events” fell into the study period

Indicate if critical “secular events” fell into the study period, e.g., significant changes in Internet resources available or “changes in computer hardware or Internet delivery resources”

	1	2	3	4	5	
subitem not at all important	◉	○	○	○	○	essential

Auswahl löschen

Does your paper address subitem 14a-i?

Copy and paste relevant sections from the manuscript (include quotes in quotation marks "like this" to indicate direct quotes from your manuscript), or elaborate on this item by providing additional information not in the ms, or briefly explain why the item is not applicable/relevant for your study

There were no such events.

14b) Why the trial ended or was stopped (early)

Does your paper address CONSORT subitem 14b? *

Copy and paste relevant sections from the manuscript (include quotes in quotation marks "like this" to indicate direct quotes from your manuscript), or elaborate on this item by providing additional information not in the ms, or briefly explain why the item is not applicable/relevant for your study

The trial did not stop early.

15) A table showing baseline demographic and clinical characteristics for each group

NPT: When applicable, a description of care providers (case volume, qualification, expertise, etc.) and centers (volume) in each group

Does your paper address CONSORT subitem 15? *

Copy and paste relevant sections from the manuscript (include quotes in quotation marks "like this" to indicate direct quotes from your manuscript), or elaborate on this item by providing additional information not in the ms, or briefly explain why the item is not applicable/relevant for your study

This table was reported in the manuscript.

15-i) Report demographics associated with digital divide issues

In ehealth trials it is particularly important to report demographics associated with digital divide issues, such as age, education, gender, social-economic status, computer/Internet/ehealth literacy of the participants, if known.

	1	2	3	4	5	
subitem not at all important	○	○	◉	○	○	essential

Auswahl löschen

Does your paper address subitem 15-i? *

Copy and paste relevant sections from the manuscript (include quotes in quotation marks "like this" to indicate direct quotes from your manuscript), or elaborate on this item by providing additional information not in the ms, or briefly explain why the item is not applicable/relevant for your study

We provided age and gender to account for these information.

16) For each group, number of participants (denominator) included in each analysis and whether the analysis was by original assigned groups

16-i) Report multiple “denominators” and provide definitions

Report multiple “denominators” and provide definitions: Report N’s (and effect sizes) “across a range of study participation [and use] thresholds” [1], e.g., N exposed, N consented, N used more than x times, N used more than y weeks, N participants “used” the intervention/comparator at specific pre-defined time points of interest (in absolute and relative numbers per group). Always clearly define “use” of the intervention.

	1	2	3	4	5	
subitem not at all important	○	◉	○	○	○	essential

Auswahl löschen

Does your paper address subitem 16-i? *

Copy and paste relevant sections from the manuscript (include quotes in quotation marks "like this" to indicate direct quotes from your manuscript), or elaborate on this item by providing additional information not in the ms, or briefly explain why the item is not applicable/relevant for your study

We provided the adherence for the intervention group and also provided for all Ns for the primary outcomes.

16-ii) Primary analysis should be intent-to-treat

Primary analysis should be intent-to-treat, secondary analyses could include comparing only “users”, with the appropriate caveats that this is no longer a randomized sample (see 18-i).

	1	2	3	4	5	
subitem not at all important	○	○	○	○	◉	essential

Auswahl löschen

Does your paper address subitem 16-ii?

Copy and paste relevant sections from the manuscript (include quotes in quotation marks "like this" to indicate direct quotes from your manuscript), or elaborate on this item by providing additional information not in the ms, or briefly explain why the item is not applicable/relevant for your study

All analyses are intention-to-treat. We calculated a sensitivity analysis for all patients who finished at least 4 modules in the intervention, but this was explicitly stated and the main analyses were intention-to-treat.

17a) For each primary and secondary outcome, results for each group, and the estimated effect size and its precision (such as 95% confidence interval)

Does your paper address CONSORT subitem 17a? *

Copy and paste relevant sections from the manuscript (include quotes in quotation marks "like this" to indicate direct quotes from your manuscript), or elaborate on this item by providing additional information not in the ms, or briefly explain why the item is not applicable/relevant for your study

"For the primary outcome PAS (t(df=110.1)=-2.22, p=.027), we found a significant time*group interaction effect, but not for the primary outcome WHO-5 (t(df=149.8)=-1.35, p=.088), as shown in table 2. Effect sizes were small to moderate for the PAS (d=-0.37, 95%-CI: -0.70; -0.04) and small for the WHO-5 (d=0.22; 95%-CI: -0.10; 0.53)."
Effect sizes of the secondary outcomes were provideed in Table 5.

17a-i) Presentation of process outcomes such as metrics of use and intensity of use

In addition to primary/secondary (clinical) outcomes, the presentation of process outcomes such as metrics of use and intensity of use (dose, exposure) and their operational definitions is critical. This does not only refer to metrics of attrition (13-b) (often a binary variable), but also to more continuous exposure metrics such as “average session length”. These must be accompanied by a technical description how a metric like a “session” is defined (e.g., timeout after idle time) [1] (report under item 6a).

	1	2	3	4	5	
subitem not at all important	○	○	○	◉	○	essential

Auswahl löschen

Does your paper address subitem 17a-i?

Copy and paste relevant sections from the manuscript (include quotes in quotation marks "like this" to indicate direct quotes from your manuscript), or elaborate on this item by providing additional information not in the ms, or briefly explain why the item is not applicable/relevant for your study

"Due to a technical problem, usage data was missing for one person in the IG. Based on data from 81 participants, the IG completed an average of 7.3 (sd=3.9) modules out of the total 12 modules, amounting to 60.8% of all modules. 26 (32.1%) participants completed the whole course, and 73 (90.1%) underwent the first four modules, which was chosen as a sensitivity analysis to assess a basic amount of engagement."

17b) For binary outcomes, presentation of both absolute and relative effect sizes is recommended

Does your paper address CONSORT subitem 17b? *

Copy and paste relevant sections from the manuscript (include quotes in quotation marks "like this" to indicate direct quotes from your manuscript), or elaborate on this item by providing additional information not in the ms, or briefly explain why the item is not applicable/relevant for your study

We calculated only Odds Ratio as an effect size, but did not provide additional absolute or relative effects since the effects were not significant anyways to keep the paper more concise.

18) Results of any other analyses performed, including subgroup analyses and adjusted analyses, distinguishing pre-specified from exploratory

Does your paper address CONSORT subitem 18? *

Copy and paste relevant sections from the manuscript (include quotes in quotation marks "like this" to indicate direct quotes from your manuscript), or elaborate on this item by providing additional information not in the ms, or briefly explain why the item is not applicable/relevant for your study

"For the BAI, a significant interaction was found at T3 (t(df=206.8)=-4.12, p < .001) with a moderate to large effect size (d=-0.60 95%-CI: -0.89; -0.32). Within-group effects (T1-T3) were large in the treatment group (d=-0.82 95%-CI: -1.05; -0.60) and small in the intervention group (d=-0.22 95%-CI: -0.39; -0.05).
For the PHQ-9, also a significant interaction was found (t(df=257.4)=-3.20, p < .001) with a small to moderate effect size (d=-0.41 95%-CI: -0.66; -0.16). Within-group effects (T1-T3) were small (d=-0.25 95%-CI: -0.42; -0.09) in the IG and showed minimal to small deterioration in the CG (d=0.15 95%-CI: -0.03; 0.34). Regarding adverse effects (NEQ), no difference was found between the groups (t=-1.14, p=.255; see Table 7 and OSM 5)."

18-i) Subgroup analysis of comparing only users

A subgroup analysis of comparing only users is not uncommon in ehealth trials, but if done, it must be stressed that this is a self-selected sample and no longer an unbiased sample from a randomized trial (see 16-iii).

	1	2	3	4	5	
subitem not at all important	○	◉	○	○	○	essential

Auswahl löschen

Does your paper address subitem 18-i?

Copy and paste relevant sections from the manuscript (include quotes in quotation marks "like this" to indicate direct quotes from your manuscript), or elaborate on this item by providing additional information not in the ms, or briefly explain why the item is not applicable/relevant for your study

"Additionally, per-protocol sensitivity analyses were conducted for both primary outcomes, including only the 73 participants (90.1%) that underwent at least the first four modules. Because no hypotheses were specified, the effects are calculated as two-tailed tests without alpha adjustment. A significant group*time interaction (t=-3.25, p=.001) was found for the PAS with a moderate effect size (d=-0.50; 95%-CI: -0.80, -0.20). Regarding the WHO-5, no significant interaction was found (t=1.80, p=.074)." This was clearly framed as a selected sample.

19) All important harms or unintended effects in each group

(for specific guidance see CONSORT for harms)

Does your paper address CONSORT subitem 19? *

Copy and paste relevant sections from the manuscript (include quotes in quotation marks "like this" to indicate direct quotes from your manuscript), or elaborate on this item by providing additional information not in the ms, or briefly explain why the item is not applicable/relevant for your study

They are reported in Table 7 and in OSM 5.

19-i) Include privacy breaches, technical problems

Include privacy breaches, technical problems. This does not only include physical “harm” to participants, but also incidents such as perceived or real privacy breaches [1], technical problems, and other unexpected/unintended incidents. “Unintended effects” also includes unintended positive effects [2].

	1	2	3	4	5	
subitem not at all important	(●)	( )	( )	( )	( )	essential

Auswahl löschen

Does your paper address subitem 19-i?

Copy and paste relevant sections from the manuscript (include quotes in quotation marks "like this" to indicate direct quotes from your manuscript), or elaborate on this item by providing additional information not in the ms, or briefly explain why the item is not applicable/relevant for your study

There were none that we know of.

19-ii) Include qualitative feedback from participants or observations from staff/researchers

Include qualitative feedback from participants or observations from staff/researchers, if available, on strengths and shortcomings of the application, especially if they point to unintended/unexpected effects or uses. This includes (if available) reasons for why people did or did not use the application as intended by the developers.

	1	2	3	4	5	
subitem not at all important	( )	(●)	( )	( )	( )	essential

Auswahl löschen

Does your paper address subitem 19-ii?

Copy and paste relevant sections from the manuscript (include quotes in quotation marks "like this" to indicate direct quotes from your manuscript), or elaborate on this item by providing additional information not in the ms, or briefly explain why the item is not applicable/relevant for your study

We did not collect any such feedback.

DISCUSSION

22) Interpretation consistent with results, balancing benefits and harms, and considering other relevant evidence

NPT: In addition, take into account the choice of the comparator, lack of or partial blinding, and unequal expertise of care providers or centers in each group

22-i) Restate study questions and summarize the answers suggested by the data, starting with primary outcomes and process outcomes (use)

Restate study questions and summarize the answers suggested by the data, starting with primary outcomes and process outcomes (use).

	1	2	3	4	5	
subitem not at all important	○	○	○	○	◉	essential

Auswahl löschen

Does your paper address subitem 22-i? *

Copy and paste relevant sections from the manuscript (include quotes in quotation marks "like this" to indicate direct quotes from your manuscript), or elaborate on this item by providing additional information not in the ms, or briefly explain why the item is not applicable/relevant for your study

"The present study compared the effectiveness of a web-based self-help iCBT for patients with panic disorder with or without agoraphobia with the care as usual over 12 weeks. In this trial, a significantly greater reduction in panic and agoraphobia symptoms was found for patients using the online intervention. No effects were found regarding the well-being of the patients. Also, no effects were found regarding the secondary hypotheses, namely daily functioning, work capacity, mental health literacy, and healthcare-related burdens. In the additional outcomes, significantly greater reductions in anxiety symptoms and depression were identified in favor of the online intervention."

22-ii) Highlight unanswered new questions, suggest future research

Highlight unanswered new questions, suggest future research.

	1	2	3	4	5	
subitem not at all important	○	○	○	◉	○	essential

Auswahl löschen

Does your paper address subitem 22-ii?

Copy and paste relevant sections from the manuscript (include quotes in quotation marks "like this" to indicate direct quotes from your manuscript), or elaborate on this item by providing additional information not in the ms, or briefly explain why the item is not applicable/relevant for your study

"This current study found only small and nonsignificant effects on well-being (d=0.22), functioning (d=0.22), and work capacity (d=0.21), which may be due to various reasons (e.g., effects do not generalize to more distal outcomes, other problems come to the surface after some symptoms are less severe anymore, lack of guidance diminishes the patients' ability to follow the program). Therefore, studies with higher statistical power are needed for reliable results. Also, modules specifically addressing these outcomes (e.g., well-being, work-related problems) could be integrated into iCBT to broaden the effects. Additionally, since insufficient adherence could also explain diminished effects in the IG, it may be worthwhile to edit the intervention to make it more engaging. It might also be necessary to integrate some form of guidance into the intervention to empower patients to follow the program more successfully."
"Another important line of future research should focus on predictors of treatment effects. Although there are some preliminary findings for moderators (alliance [65] and impairment [66]), further research is warranted to enable clinicians to design more effective treatment courses."

20) Trial limitations, addressing sources of potential bias, imprecision, and, if relevant, multiplicity of analyses

20-i) Typical limitations in ehealth trials

Typical limitations in ehealth trials: Participants in ehealth trials are rarely blinded. Ehealth trials often look at a multiplicity of outcomes, increasing risk for a Type I error. Discuss biases due to non-use of the intervention/usability issues, biases through informed consent procedures, unexpected events.

	1	2	3	4	5	
subitem not at all important	○	○	○	◉	○	essential

Auswahl löschen

Does your paper address subitem 20-i? *

Copy and paste relevant sections from the manuscript (include quotes in quotation marks "like this" to indicate direct quotes from your manuscript), or elaborate on this item by providing additional information not in the ms, or briefly explain why the item is not applicable/relevant for your study

"Fourth, as in any psychotherapeutic trial comparing an intervention group versus a waiting group, the participants were not blinded towards their group allocation."
Regarding the multiplicity of outcome, we calculated appropriate adjustments for multiple testing via Bonferroni-Holm: "The p-values were adjusted for two tests according to Bonferroni-Holm adjustment", "Secondary confirmatory outcomes were calculated only after success in the primary analysis to prevent alpha inflation...", "For the four secondary outcomes, Bonferroni-Holm adjustment was conducted as well."

21) Generalisability (external validity, applicability) of the trial findings

NPT: External validity of the trial findings according to the intervention, comparators, patients, and care providers or centers involved in the trial

21-i) Generalizability to other populations

Generalizability to other populations: In particular, discuss generalizability to a general Internet population, outside of a RCT setting, and general patient population, including applicability of the study results for other organizations

	1	2	3	4	5	
subitem not at all important	○	○	○	○	○	essential

Does your paper address subitem 21-i?

Copy and paste relevant sections from the manuscript (include quotes in quotation marks "like this" to indicate direct quotes from your manuscript), or elaborate on this item by providing additional information not in the ms, or briefly explain why the item is not applicable/relevant for your study

The generalizability was assessed via the PRECIS-2 tool and was discussed in the limitations: "Second, this study has some deviations from usual care in Germany, which were assessed via the PRECIS-2 tool ([67]; see OSM 7): Since many patients were recruited via university and social media and not from a mental health provider, this study may only partly reflect routine care. This is also indicated by the finding that our sample was predominantly female (OR=6.1), with a substantially higher percentage than in a representative sample from the US population (OR=2.0; [68]). This may not be too surprising since women show generally higher levels of help-seeking [69], but it could also be due to the recruitment strategy, which is a deviation from the usual recruitment. Other deviations include the structured diagnostic interview and the two follow-up assessments, which may impede external validity. However, other aspects of the trial (e.g., eligibility, delivery, primary hypotheses) were designed with high external validity, altogether indicating a pragmatic (routine care) trial as assessed via PRECIS-2."

21-ii) Discuss if there were elements in the RCT that would be different in a routine application setting

Discuss if there were elements in the RCT that would be different in a routine application setting (e.g., prompts/reminders, more human involvement, training sessions or other co-interventions) and what impact the omission of these elements could have on use, adoption, or outcomes if the intervention is applied outside of a RCT setting.

	1	2	3	4	5	
subitem not at all important	○	○	◉	○	○	essential

Auswahl löschen

Does your paper address subitem 21-ii?

Copy and paste relevant sections from the manuscript (include quotes in quotation marks "like this" to indicate direct quotes from your manuscript), or elaborate on this item by providing additional information not in the ms, or briefly explain why the item is not applicable/relevant for your study

This was also discussed with the PRECIS-2 tool: "Since many patients were recruited via university and social media and not from a mental health provider, this study may only partly reflect routine care. This is also indicated by the finding that our sample was predominantly female (OR=6.1), with a substantially higher percentage than in a representative sample from the US population (OR=2.0; [68]). This may not be too surprising since women show generally higher levels of help-seeking [69], but it could also be due to the recruitment strategy, which is a deviation from the usual recruitment. Other deviations include the structured diagnostic interview and the two follow-up assessments, which may impede external validity. However, other aspects of the trial (e.g., eligibility, delivery, primary hypotheses) were designed with high external validity, altogether indicating a pragmatic (routine care) trial as assessed via PRECIS-2."

OTHER INFORMATION

23) Registration number and name of trial registry

Does your paper address CONSORT subitem 23? *

Copy and paste relevant sections from the manuscript (include quotes in quotation marks "like this" to indicate direct quotes from your manuscript), or elaborate on this item by providing additional information not in the ms, or briefly explain why the item is not applicable/relevant for your study

DRKS00023800 https://drks.de/search/en/trial/DRKS00023800

24) Where the full trial protocol can be accessed, if available

Does your paper address CONSORT subitem 24? *

Cite a Multimedia Appendix, other reference, or copy and paste relevant sections from the manuscript (include quotes in quotation marks "like this" to indicate direct quotes from your manuscript), or elaborate on this item by providing additional information not in the ms, or briefly explain why the item is not applicable/relevant for your study

"Study protocol: The study protocol was published during participant enrolment (https://doi.org/10.1016/j.invent.2022.100584)"

25) Sources of funding and other support (such as supply of drugs), role of funders

Does your paper address CONSORT subitem 25? *

Copy and paste relevant sections from the manuscript (include quotes in quotation marks "like this" to indicate direct quotes from your manuscript), or elaborate on this item by providing additional information not in the ms, or briefly explain why the item is not applicable/relevant for your study

"The study was funded by a European Regional Development Fund awarded to Selfapy. The Professorship for Adult Clinical Psychology and Psychotherapy at Osnabrück University was asked by Selfapy to independently evaluate its product to be assessed to the best of our knowledge and following the standards of good scientific practice. Selfapy made suggestions for the selection of survey instruments in the study's design but had no influence on the data collection, analysis, and interpretation or production of the resulting publications."

X27) Conflicts of Interest (not a CONSORT item)

X27-i) State the relation of the study team towards the system being evaluated

In addition to the usual declaration of interests (financial or otherwise), also state the relation of the study team towards the system being evaluated, i.e., state if the authors/evaluators are distinct from or identical with the developers/sponsors of the intervention.

	1	2	3	4	5	
subitem not at all important	◉	○	○	○	○	essential

Auswahl löschen

Does your paper address subitem X27-i?

Copy and paste relevant sections from the manuscript (include quotes in quotation marks "like this" to indicate direct quotes from your manuscript), or elaborate on this item by providing additional information not in the ms, or briefly explain why the item is not applicable/relevant for your study

"None declared." There were no conflict of interests - the study team is not associated with Selfapy and was not involved in the creation of Selfapy.

About the CONSORT EHEALTH checklist

As a result of using this checklist, did you make changes in your manuscript? *

- ○ yes, major changes
- ◉ yes, minor changes
- ○ no

What were the most important changes you made as a result of using this checklist?

I think there were no "most important changes", but only small changes here and there.

How much time did you spend on going through the checklist INCLUDING making changes in your manuscript *

Altogether, this would be 6 hours

As a result of using this checklist, do you think your manuscript has improved? *

- (x) yes
- ( ) no
- ( ) Sonstiges:

Would you like to become involved in the CONSORT EHEALTH group?

This would involve for example becoming involved in participating in a workshop and writing an "Explanation and Elaboration" document

- ( ) yes
- (x) no
- ( ) Sonstiges:

Auswahl löschen

Any other comments or questions on CONSORT EHEALTH

It would have been great to get access to the checklist before the peer-review-process was finished. It feels like an additional peer-review round now, which could have been cleared already in an earlier round if we had access to the manuscript.

STOP - Save this form as PDF before you click submit
To generate a record that you filled in this form, we recommend to generate a PDF of this page (on a Mac, simply select "print" and then select "print as PDF") before you submit it.

When you submit your (revised) paper to JMIR, please upload the PDF as supplementary file.

Don't worry if some text in the textboxes is cut off, as we still have the complete information in our database. Thank you!

Final step: Click submit !
Click submit so we have your answers in our database!

Senden

Alle Eingaben löschen

Geben Sie niemals Passwörter über Google Formulare weiter.

Dieser Inhalt wurde nicht von Google erstellt und wird von Google auch nicht unterstützt. - Nutzungsbedingungen - Datenschutzerklärung

Does this form look suspicious? Bericht

Google Formulare
