# Supplementary material for: Evaluation of an Online-Based Self-Help Program for Patients With Panic Disorder: Randomized Controlled Trial
Source: J Med Internet Res. 2025 Apr 2;27:e54062. doi: 10.2196/54062 (PMC12004017; doi:10.2196/54062)

# OSM

## OSM 1

*Linear mixed model and effect sizes for the PAS – Imputed data and Completer T3-T1*

|  | | **Group*Time** | | | | |
| --- | --- | --- | --- | --- | --- | --- |
| **Imputation/Group** | | ***t*** | ***df*** | ***p* (two-sided)** | **Interaction effect (95%-CI)** | |
| T3 Completer | | | | | |  |
|  | -2.25 | | 129 | .026* | -0.34 (-0.64; -0.04) |  |
| LOCF | | | | | |  |
|  | -2.67 | | 154 | .008** | -0.37 (-0.64; -0.10) |  |
| BOCF | | | | | |  |
|  | -2.30 | | 154 | .023* | -0.30 (-0.56; -0.04) |  |
| J2R | | | | | |  |
|  | -1.10 | | 3171.26 | .272 | -0.30 (-0.83; 0.23) |  |

*Note*. * indicates p ≤ .05, ** indicates p ≤ .01, *** indicates p ≤ .001. LOCF, Last-observation-carried-forward; BOCF, Baseline-observation-carried-forward; J2R, Reference-based-multiple imputation ("jump-to-reference").

## OSM 2

*T3 Completer values over time for the PAS*
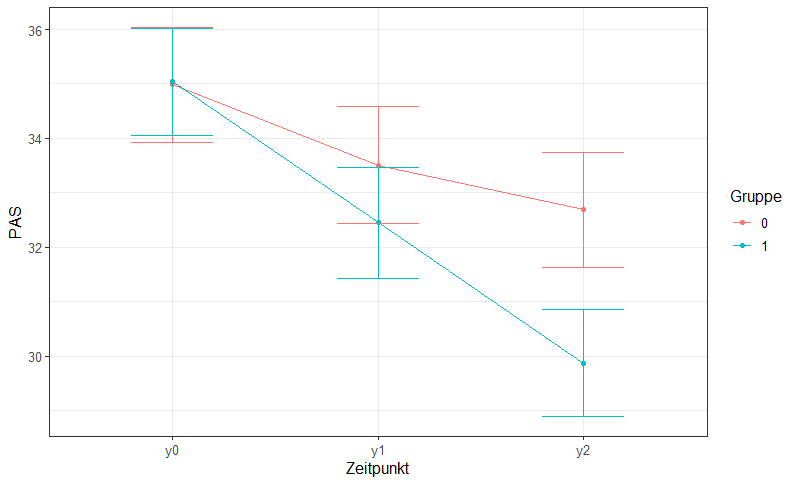


## OSM
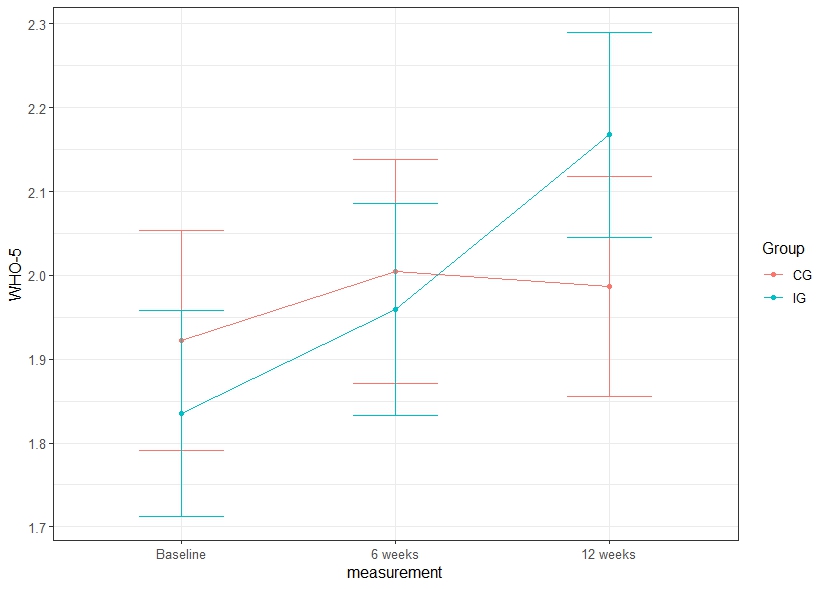
3

*T3 Completer values over time for the WHO-5*

## OSM 4

*Linear mixed model and effect sizes for the WHO-5 – Imputed data and Completer T3-T1*

|  | **Group*Time** | | | |
| --- | --- | --- | --- | --- |
| **Imputation/Group** | ***t*** | ***df*** | ***p* (two-sided)** | **Interaction effect  (95%-CI)** |
| T3 Completer | | | | |
|  | 1.50 | 125 | .136 | 0.27 (-0.08; 0.61) |
| LOCF | | | | |
|  | 1.62 | 154 | .107 | 0.24 (-0.05; 0.52) |
| BOCF | | | | |
|  | 1.54 | 154 | .125 | 0.22 (-0.06; 0.51) |
| J2R | | | | |
|  | 1.27 | 53021.02 | .205 | 0.20 (-0.11; 0.50) |

*Note*. * indicates p ≤ .05, ** indicates p ≤ .01, *** indicates p ≤ .001. LOCF, Last-observation-carried-forward; BOCF, Baseline-observation-carried-forward; J2R, Reference-based-multiple imputation ("jump-to-reference").

## OSM 5

*All negative effects according to groups*

| **Negative effect** | **Frequency Control Group** | **Frequency Intervention Group** |
| --- | --- | --- |
| I had more problems with my sleep | 32 (43.2%) | 15 (18.3%) |
| I felt like I was under more stress | 22 (29.7%) | 17 (20.7%) |
| I experienced more anxiety | 23 (31.1%) | 11 (13.4%) |
| I felt more worried | 25 (33.8%) | 11 (13.4%) |
| I felt more dejected | 13 (17.6%) | 10 (12.2%) |
| I experienced more hopelessness | 8 (10.8%) | 5 (6.1%) |
| I experienced lower self-esteem | 8 (10.8%) | 2 (2.4%) |
| I lost faith in myself | 9 (12.2%) | 6 (7.3%) |
| I felt sadder | 15 (20.3%) | 7 (8.5%) |
| I felt less competent | 9 (12.2%) | 3 (3.7%) |
| I experienced more unpleasant feelings | 25 (33.8%) | 14 (17.1%) |
| I felt that the issue I was looking for help with got worse | 4 (5.4%) | 7 (8.5%) |
| Unpleasant memories resurfaced | 23 (31.1%) | 38 (46.3%) |
| I became afraid that other people would find out about my treatment | 8 (10.8%) | 3 (3.7%) |
| I started feeling ashamed in front of other people because I was having treatment | 3 (4.1%) | 2 (2.4%) |
| I stopped thinking that things could get better | 6 (8.1%) | 6 (7.3%) |
| I started thinking that the issue I was seeking help for could not be made any better | 4 (5.4%) | 6 (7.3%) |
| I stopped thinking help was possible | 10 (13.5%) | 6 (7.3%) |
| I think that I have developed a dependency on my treatment | 5 (6.8%) | 2 (2.4%) |
| I did not always understand my treatment | 5 (6.8%) | 6 (7.3%) |
| I did not have confidence in my treatment | 1 (1.4%) | 4 (4.9%) |
| I felt that the treatment did not produce any results | 3 (4.1%) | 10 (12.2%) |
| I felt that my expectations for the treatment were not fulfilled | 2 (2.7%) | 11 (13.4%) |
| I felt that the quality of the treatment was poor | 1 (1.4%) | 1 (1.2%) |
| I felt that the treatment did not suit me | 1 (1.4%) | 7 (8.5%) |
| I felt that the treatment was not motivating | 1 (1.4%) | 3 (3.7%) |

## OSM 6

*Diagnoses*

|  | **Intervention Group  (*N* = 81)** | **Control Group  (*N* = 73)** | **Total  (*N* = 154)** | **Fisher’s exact test *p*-value** |
| --- | --- | --- | --- | --- |
| Generalized anxiety disorder current | 31 (38.3%) | 17 (23.3%) | 48 (31.2%) | .056 |
| Generalized anxiety disorder past | 3 (3.7%) | 6 (8.2%) | 9 (5.8%) | .309 |
| Panic disorder current | 27 (33.3%) | 32 (43.8%) | 59 (38.3%) | .189 |
| Panic disorder with agoraphobia | 54 (66.7%) | 41 (56.2%) | 96 (61.7%) | .189 |
| Agoraphobia current | 54 (66.7%) | 41 (56.2%) | 95 (61.7%) | .189 |
| Agoraphobia past | 0 (0%) | 0 (0%) | 0 (0%) | NA |
| Major depressive disorder current | 8 (9.8%) | 1 (1.4%) | 9 (5.8%) | .080 |
| Major depressive disorder past | 15 (18.5%) | 21 (28.8%) | 36 (23.4%) | .182 |
| Alcohol abuse past | 0 (0%) | 0 (0%) | 0 (0%) | NA |
| Substance abuse past | 2 (2.5%) | 1 (1.4%) | 3 (1.9%) | 1.000 |
| Dysthymia current | 6 (7.4%) | 5 (6.8%) | 11 (7.1%) | 1.000 |
| Dysthymia past | 3 (3.7%) | 2 (2.7%) | 5 (3.2%) | 1.000 |
| Separation anxiety current | 19 (23.5%) | 16 (21.9%) | 35 (22.7%) | .850 |
| Separation anxiety past | 2 (2.5%) | 5 (6.8%) | 7 (4.5%) | .257 |
| Social phobia current | 29 (35.8%) | 11 (15.1%) | 40 (26.0%) | .005** |
| Social phobia past | 0 (0.0%) | 4 (5.5%) | 4 (2.6%) | .048* |
| Obsessive-compulsive disorder current | 1 (1.2%) | 2 (2.7%) | 3 (1.9%) | .604 |
| Obsessive-compulsive disorder past | 5 (6.2%) | 1 (1.4%) | 6 (3.9%) | .213 |
| Bodily dysmorphic disorder current | 4 (4.9%) | 0 (0.0%) | 4 (2.6%) | .122 |
| Bodily dysmorphic disorder past | 2 (2.5%) | 0 (0.0%) | 2 (1.3%) | .498 |
| Post-traumatic stress disorder | 10 (12.3%) | 5 (6.8%) | 15 (9.7%) | .251 |
| Somatoform disorder current | 1 (1.2%) | 3 (4.1%) | 4 (2.6%) | .346 |
| Somatoform disorder past | 1 (1.2%) | 4 (5.5%) | 5 (3.2%) | .191 |
| Illness anxiety disorder current | 14 (17.3%) | 11 (15.1%) | 25 (16.2%) | .828 |
| Illness anxiety disorder past | 1 (1.2%) | 1 (1.4%) | 2 (1.3%) | 1.000 |
| Anorexia current | 0 (0.0%) | 1 (1.4%) | 1 (0.7%) | .474 |
| Anorexia past | 7 (8.6%) | 4 (5.5%) | 11 (7.1%) | .540 |
| Bulimia current | 0 (0%) | 0 (0%) | 0 (0%) | NA |
| Bulimia past | 1 (1.2%) | 1 (1.4%) | 2 (1.3%) | 1.000 |
| Binge eating disorder current | 1 (1.2%) | 0 (0.0%) | 1 (0.6%) | 1.000 |
| Binge eating disorder past | 1 (1.2%) | 2 (2.7%) | 3 (1.9%) | .604 |
| Borderline Personality disorder current | 0 (0.0%) | 1 (1.4%) | 1 (0.6%) | .474 |

*Note.* * indicates *p ≤* .05, ** indicates *p ≤* .01, *** indicates *p ≤* .001. Past diagnoses were only assessed if no current diagnosis was given.

## OSM 7

*The PRECIS-2 tool*

| **Domain** | **Score** | **Rationale** |
| --- | --- | --- |
| Eligibility Criteria | 5 | In comparison to usual care, similar restrictions apply. Due to the German regulations, all restrictions apply for the internet-based CBT (i.e., exclusion of severe depression, old age, or a history of psychosis). |
| Recruitment | 1 | Due to the targeted recruitment via social media and email listservs, this is mainly an explanatory approach. |
| Setting | 5 | The setting is the same as in usual care. |
| Organisation | 3 | In comparison to usual care, diagnostic interviews are conducted by psychologists, instead of diagnosis by general practitioners. |
| Flexibility (delivery) | 5 | Delivery flexibility is the same as in usual care. |
| Flexibility (adherence) | 4 | With the excemption of the diagnostic interview, which was conducted neutrally in respect to treatment adherence, there are not differences to care as usual. |
| Follow-up | 3 | There are two follow-up-assessments at 6 weeks and 12 weeks, which are short and can be conducted flexibly online. |
| Primary outcome | 5 | The primary outcome is highly relevant for the patients. |
| Primary analysis | 5 | Analysis was conducted via intention-to-treat. |


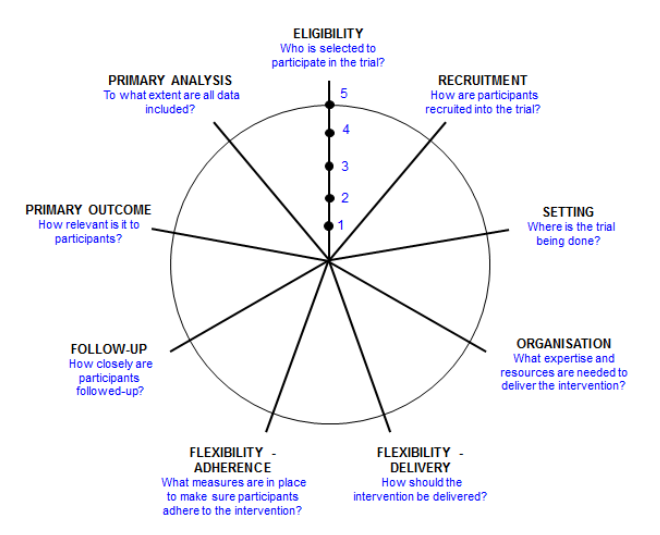

Supplement: Multimedia Appendix 2 [file jmir_v27i1e54062_app2.docx]
